# Modes of embodiment for an immersed experience in museums: The Royal Tank Museum

**DOI:** 10.1371/journal.pone.0317402

**Published:** 2025-01-29

**Authors:** Lama B. Abuhassan, Shatha R. Malhis

**Affiliations:** 1 Department of Digital Film Design Technology, University of Petra, Amman, Jordan; 2 Department of Interior Design, University of Petra, Amman, Jordan; University of California Santa Cruz, UNITED STATES OF AMERICA

## Abstract

This study examines how exhibits at the Royal Tank Museum (RTM) engage visitors through bodily experiences. The goal is to understand RTM’s visitor interaction and engagement. This research fills a gap in the literature on Jordanian museums by documenting some experiential characteristics of museum exhibits and establishing a framework for evaluating embodied museum encounters. A detailed RTM episode analysis, online survey, visitor behavior observations, structured interviews, and a comprehensive questionnaire are used. Al-Karameh Hall offers embodied experiences using sensory and physical tools, while Jerusalem Hall and the Battle of Al-Latrun fall short due to fractured narratives and inadequate sensory engagement. According to the study, boosting the clarity of narratives, adding personal belongings, and strengthening the spatial connection between exhibitions and their surrounds can greatly enhance visitors’ embodied experiences.

## 1.Introduction

Over the past three decades, museum design has adopted an interpretative paradigm whereby visitors’ interactive experiences are central. Audience research that focuses on the museum as an experience highlights the use of new technology to produce high-quality interactive environments [1–4].

Embodied experiences in museums and memorials can have a profound impact on users, often more so than traditional museums. While traditional museums typically rely on a visual and intellectual approach to presenting information with exhibits designed primarily to inform and educate, embodied experiences focus on engaging the user’s physical and emotional senses, creating a more immersive and impactful experience [4–8].

**Fig 1:**
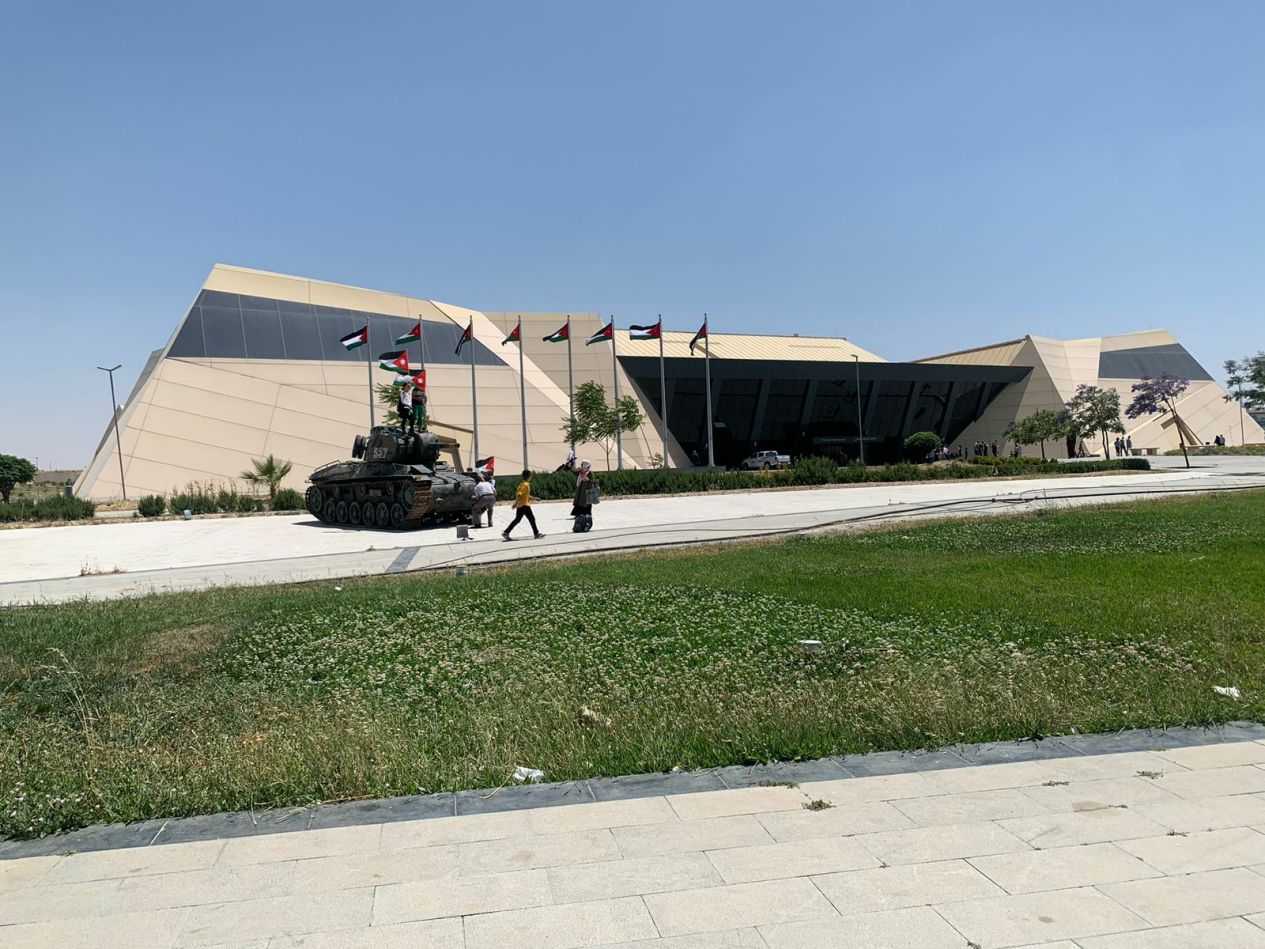
The Royal Tank Museum (RTM), Amman- Jordan.

This study focuses on the Royal Tank Museum (RTM) in Amman, Jordan with its 150 tanks. It is a museum that narrates stories of patriotism and sacrifice of the Jordanians under the leadership of the Royal Hashemite leader. The museum provides a wealth information and displays spread across an area of 20,000 m^2;^ it tells stories of local and global wars, and heroes in various tank battles (Fig 1).

Certain publications discuss the subject of museums in Jordan, with some focusing on environmental, aesthetic, and architectural aspects of museums, with calls to examine the emotional engagement prompted by museum visits [9], advocating for virtual museums to enhance tourist attractions [10], or discussing the architectural reuse of spaces with a focus on deliberate design in historic locations [11].

As for RTM in particular, there is a single study by Malhis et al. (2021), which focused on architectural and curatorial methods without examining visitors’ embodied experiences [12]. This study examines the RTM’s experiential and interactive exhibitions to fill this gap. It analyzes the interactive nature of RTM and the effects of its displays throughout its fifteen sections (episodes). It establishes a framework for this research by defining a collection of comprehensive study areas necessary to understand museum experiences. This research begins by addressing the following question.

Which tools can address the experiential aspects of museum visits, whether traditional or embodied? Which tools are essential for understanding the individual-environment interaction at RTM, and how can they be integrated methodologically?

The research begins by explaining the theoretical basis for embodied experiences, as stated by various theorists, to help visitors engage with their environment. It stresses how the body and physical environment interact and how certain tools affect the emotional and cognitive museum experience. The study seeks to identify the tools employed to narrate the museum’s stories and to underscore their contextual relevance. After establishing the theoretical concepts, the subsequent part of the research uses theoretical discussions to formulate methodological tools.

The third section introduces RTM specifically and describes the different sections of the museum (episodes) and their sequential arrangement as designed by the museum curators. It also engages in an online survey to collect vivid memories from locals relating to the moments in Jordanian military history, the events, battles, and characters they feel most connected to. Then, the research delves in analytical examinations by presenting: (a) the distribution and integration of thematic tools as identified by the literature within RTM episodes and spaces, using descriptive analyses to conceptualize RTM settings; (b) addressing the experiential dimension of RTM’s episodes, utilizing visitors’ observations; (c) analyzing the interactions between visitors and the physical environment through data gathered from a structured and thematic questionnaire.

The final section evaluates the data and discusses RTM-specific findings, theoretical and methodological implications, and the contribution of this study to museum research in the man-environment paradigm.

## 2.Theoretical concepts and tools

The following section introduces the theoretical concepts and tools that are essential to the progress of this research.

### 2.1 Embodiment and embodiment modes

Understanding embodied experiences in museums involves recognizing the role of affective atmospheres—emotional, sensory, and atmospheric conditions—in influencing visitor behavior and emotions [6,13–19]. Research highlights how these atmospheres are shaped by various tools like text, light, sensory stimuli, and technology, and how these are framed within narratives to enhance the museum experience [1,2,5,20–22].

According to Merleau-Ponty embodiment is the body’s relationship with its environment, with a distinction drawn between the material body (physical, objective) and the felt body (subjective, involving sensations and emotions) [23]. This distinction emphasizes the body’s dual role as an observer and a participant in forming experiences, emphasizing the interdependence of corporeality (physical presence) and consciousness. Corporeality is the body’s interaction with the world, while consciousness is the felt experiences and emotional responses [23,24]. The corporeal experience requires physical and sensory modes, while consciousness requires emotional and cognitive modes, according to research on embodiment.

**Fig 2:**
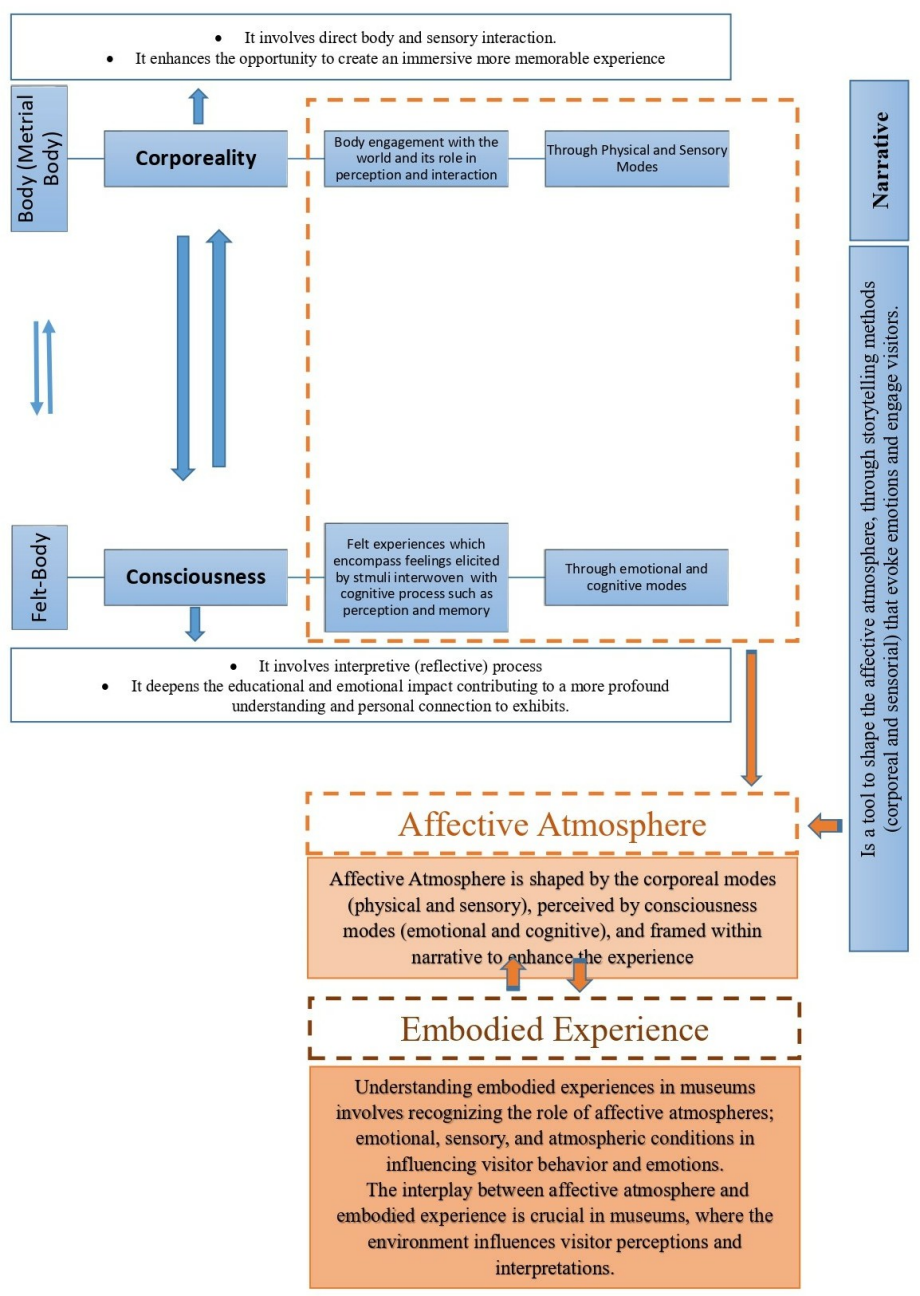
The concept of embodiment illustrating the interaction between the body and the environment in museums.

The interplay between affective atmosphere and embodied experience is crucial in museums, where the environment influences visitor perceptions and interpretations [1,5,20–22,25,26]. This reciprocal relationship demonstrates how narrative and atmosphere create engaging and meaningful museum experiences, emphasizing the need to embody content and themes holistically. Fig 2 illustrates the concept of embodiment.

Scholars and researchers use a multidisciplinary approach to assess the embodied experience in museums, drawing on fields such as museum studies, anthropology, psychology, and design research. These methods include qualitative approaches such as ethnographic research, interviews, and participant observation, as well as quantitative methods such as surveys and questionnaires. Table 1 summarizes the different methods used by scholars in assessing embodied experience in museums.

**Table 1:** A summary of different methods used to assess embodied experience in museums.

Scholars	Methods Used for Assessing Embodied Experience in Museums
**Isbister, Höök, Sharp, and Laaksolahti** **[27]**	Developed and tested the Sensual Evaluation Instrument, a nonverbal tool for real-time self-reporting of affective responses, using tactile objects for intuitive and immediate feedback.
**Kenderdine** **[28]**	Analysis of virtual embodiment, immersion, and performance through extensive user evaluation survey for Place Hampi.
**Wineman & Peponis** **[29]**	Utilized behavioral observations and spatial layout analysis, focusing on visibility polygons and accessibility, to study visitor movement patterns and exhibit engagement.
**Patrizia Schettino** **[7]**	Utilized ’embodied constructivist GTM digital ethnography in situ,’ combining grounded theory and digital ethnography for in-situ observations in immersive environments, focusing on culturally diverse visitor interpretations.
**Naspetti et al.; Sparrow** **[30,31]**	Use of technological tools such as sensors and eye-tracking devices to measure visitors’ physiological responses and behaviors.
**Tzortzi & Tzortzi** **[8]**	Employed spatial layout analyses and space syntax theory; analyzed spatial organization through graphical representations to understand sensory and interactive aspects.
**Perry et al.** **[32]**	Employed a phenomenological approach with questionnaires to investigate audience experiences in digitally augmented exhibitions, gathering both quantitative and qualitative data.
**Malhis et al.** **[12]**	Used a multi-layered approach combining spatial and visual analyses with ethnographic methods like observations and semi-structured interviews to understand spatial organization and visitor experiences.

### 2.2 Narrative as a tool

Narrative shapes museum experiences by evoking emotions and engaging visitors [33]. Merleau-Ponty emphasizes the role of narrative in shaping self and history by weaving time and experience into a story [23]. Tricia Austin promotes narrativity as an innovative method to create narrative environments, emphasizing plot and agency to promote embodied perception and intellectual transformation [34]. Pioneers like Sarah Kenderdine and Stephen Greenberg emphasize the importance of immersive narratives in making exhibits engaging, advocating for a multisensory approach to achieve embodied representation [2,7]. Austin adds that narrative environments should combine form, color, light, and content (words, images) to engage visitors [34].

The narrative can be direct, guiding visitors with a script, or suggestive, encouraging active engagement, often combining both for a richer experience [34]. Austin also discusses how design enhances sensory and physical experiences by offering multiple interpretations. Adding cultural motifs and spatial arrangements to narratives improves comprehension and emotional connection, according to Keunhye Lee [35].

Researchers have introduced the concept of the “immersive narrative environment” to highlight the relationship between corporeality and consciousness. Merleau-Ponty’s corporeality emphasizes the body’s sensory interaction with the environment, while Schmitz’s felt-body concept (consciousness) emphasizes emotion and cognition. This suggests that a well-integrated narrative promotes both physical and intellectual emotions for a complete embodied experience. These theoretical concepts and their physical and sensory tools are discussed below.

#### 2.2.1 Corporeal experience (material-body)

The concept of embodiment emphasizes the body’s role in perception and engagement. It implies that bodies shape interactions and experiences through physical interaction with the world and via sensorial channels for receiving and interpreting environmental stimuli.

*2*.*2*.*1*.*1 Physical Tools-Physical Experience*. The corporeal experience can be achieved through physical embodiment; this refers to the ways in which visitors physically interact with the exhibits and museum space, whether through physical interaction like hands-on activities and hands-on objects, reading some text, or indirect physical interaction relating, for example, to museum location, staging and theatrical aspects, and the display of personal belongings. Physical engagement can create more embodied experiences and evoke museum memories.

Hands-on objects:

This study defines “hands-on” as museum visitors physically engaging with exhibits or interactive tasks. Hands-on interaction with the museum environment lets visitors shape a narrative. Hands-on elements make museum visits more immersive. Interactive exhibits allow visitors to touch, manipulate, and move objects, deepening their understanding. This active participation transforms museum visits from passive observation to exploratory and educational adventures, surpassing reading descriptions or viewing objects behind glass.

Videos, soundscapes, and virtual reality (VR) installations enhance immersive experiences of hands-on objects; these technologies can transport visitors to different times and places, enhancing presence and immersion and providing a multi-sensory journey through the museum’s narrative landscape. Table 2 gives examples of ways visitors interact with exhibits.

**Table 2:** Examples of hands-on experiences in some museums.

Museum/Exhibition	Location	Year	Hands-on Experience Description
**Denver Museum of Nature & Science**	Denver, Colorado, USA	Opened in 1900, ongoing exhibits	Known for its hands-on science exhibits, allowing visitors to engage directly with scientific concepts and phenomena.
**The Henry Ford Museum**	Dearborn, Michigan, USA	Opened in 1929, ongoing exhibits	Offers a variety of interactive learning experiences across its attractions, catering to all ages.
**Intrepid Sea, Air & Space Museum**	New York, NY, USA	Opened in 1982, ongoing exhibits	Features numerous interactive exhibits, including a real-life helicopter, enhancing visitor engagement with military and maritime history.
**"In Praise of Shadows" at the V&A Museum**	London, UK	2009	Visitors used hand-powered LED torches to explore exhibits in the dark, allowing for personal discovery.
**"Shrek" Museographic Experience**	Various locations worldwide Ex. London	N/A (varies by location).	Children engaged in the narrative by delivering objects to characters, enhancing the interactive experience.
**Paradox Museum**	Various locations worldwide. Ex. Paris	N/A (varies by location)	Encourages hands-on exploration through exhibits that respond to visitor presence and allow manipulation of scientific phenomena.

Text

Text affects visitors’ emotions and experiences by connecting them on an embodied level. Merleau-Ponty claims that words have a "motor physiognomy", causing behavioral responses that shape our perceptions and actions, enriching the atmospheric encounter with text and shaping unique experiences [23]. Based on this, Foroni and Semin and Oosterwijk et al. explore language’s embodied nature and its profound impact on our worldview [36,37]. Foroni and Semin argue that language, especially when conveying emotions, is an embodied stimulus that shapes physical readiness and experience [38].

Thus, the presentation and choice of words in a museum can greatly impact the emotional climate and visitor experience, demonstrating the power of text to engage visitors cognitively and physically. Table 3 presents the role of text in museum design.

**Table 3:** Different ways that text can evoke emotions in museums.

Ways text can evoke emotions in museums	Example
**Use of narrative**	The Natural History Museum in London uses a dramatic text to tell the story of a dinosaur’s discovery and excavation, creating a sense of awe and wonder.
**Use of emotive language**	The 9/11 Memorial and Museum in New York uses personal stories and quotes from victims, survivors, and rescue workers with emotive language to create a powerful and emotional atmosphere. The use of first-person accounts and emotive language, such as "lost," "survived," and "remembered," greatly enhances visitors’ emotional experiences and helps them to connect with the events of 9/11 on a personal level [39]
**Enhancing embodied experiences**	The British Museum’s Parthenon gallery provides information on the myths and legends associated with the sculptures, encouraging visitors to engage with the objects in a more imaginative and embodied way.
**Innovative use of text for a spatial relationship**	The Brickworks Museum in Zehdenick uses text at 100 degrees to create a more spatial relationship with the circular Kiln to actively embody the experience of high temperature [1,40].
**Creating a sense of atmosphere**	The Apartheid Museum in South Africa uses the words “whites” and “non-whites” to immerse visitors in the reality of apartheid, making it a tangible and embodied experience [34].

Location

Location in museums creates a conceptual bridge between the past and present, allowing visitors to connect with the memories and emotions of a specific time and place. As Greenberg suggests, real events in their real physical settings and authentically narrated in original diaries or other artifacts enhance the narrative experience. Visitors can relate to the story more deeply due to this integrated and emotional experience [5]. The physical experience of a historic building, with its associated smells and tactile sensations tied to past uses, heightens interest in a more sensory mode of engagement with the material world, as in the case of Govan Old Church in Glasgow; the noise, smell, and scale of the building are all recreated to dramatize the experience and bring the story to life for visitors [26].

Staging

Exhibition staging turns museums into theaters with dynamic displays. As shown in Table 4, Aronson from Columbia University’s School of the Arts connects architecture and theater’s focus on reshaping space and conveying information to evoke emotion [41].

**Table 4:** The application of staging techniques in museum exhibitions.

Museum/Exhibition	Location	Year	Staging Description
**"Spectres: When Fashion Turns Black" at the V&A Museum**	London, UK	2005	Utilized theatrical devices to explore themes of transformation, guiding visitors’ vision through the space.
**"The World as a Stage" at Tate Modern**	London, UK	2007	Structured in three acts with installations using theatrical elements like scenery and props.
**"The Enchanted Palace" at Kensington Palace**	London, UK	2011	Employed theatrical conventions with installations and live performances for playful visitor engagement. Several rooms, and each room tells a story of a princess, each room was named after its story, for example, the Room of Royal Sorrows, the Room of Enlightenment, the Seat of Power, the Room of Flight, and the Room of Royal Secrets.

Modern museums use scenic design, digital backdrops, and live performances to create immersive experiences. Building on theatrical traditions, museum designers use illusions and props to engage visitors as actors, directors, and witnesses. Stagecraft elements like scene-setting, mechanical effects, precise lighting, soundscapes, and wax figures add realism and depth to the museum’s atmosphere. In these carefully curated spaces, every object and prop is placed to encourage active engagement with the exhibits [33,42]. More examples in Table 4.

Personal Belongings

Personal belongings can evoke strong memories of people and events. They can be used in museums and memorials to tell stories and depict historical figures or ordinary people during significant events. These objects allow visitors to imagine their former owners and their experiences, which increases empathy and visitors’ understanding of history. In a museum, personal objects gain new meaning and allow visitors to emotionally connect to the events and people they represent, making history more relatable. Table 5 gives examples of how personal belongings can shape narratives.

**Table 5:** The role of personal belongings in creating narratives.

*Personal items*	*Sense of*	*Revealing*
Soldier’s pair of shoes at the Australian Memorial	Empathy	The toll of the tragedy
The diary of Anne Frank and her comb- Anne Frank Museum	Empathy	WWII’s brutality
Ruins of a barbershop chair- Eastern State Penitentiary in the USA and in the Galleries of Justice in the UK	Empathy	Harsh conditions of past prison
Queen Elizabeth’s crown and wedding dress- in the Jewel House at the Tower of London.	Nostalgia	Historical moment

Empathy refers to the ability to understand and share the feelings of others.

Nostalgia is a sentimental longing or wistful affection for the past, typically for a period or place with happy personal associations.

2.2.1.2 Sensory Experience

Sensory Tools

Sensory embodiment in museums includes the many ways visitors interact with exhibits beyond touch and sight from hands-on objects and textual descriptions. Utilizing sensory tools to animate exhibits provides a holistic experience through sight, sound, smell, and texture. To connect visitors to the past, museums may use soundscapes or aromas to create a specific atmosphere or time period.

Pallasmaa, Holl et al., Waterton & Dittmer, and Anderson emphasize the importance of sensory experiences in architectural and memorial contexts [6,15,43,44]. These perspectives show how sensory engagement shapes nuanced understanding of spaces and narratives. Table 6 indicates scholars’ recognition of sensory experiences.

**Table 6:** The significance of sensory experiences as highlighted by scholars.

Scholar	Key Contributions
Merleau-Ponty [23]	Advocates that sensory experiences are active and dynamic, involving the whole body, and that our perceptions of the world are shaped by our body’s actions and movements.
Holl et al. [44]	Suggests that designers can utilize sensory cues like light, sound, and materials to create atmospheric experiences that resonate emotionally with visitors, evoking personal memories and feelings.
Juhani Pallasmaa [43]	Emphasizes that the sensory experience of architecture is holistic and embodied, advocating for an integrated approach to the senses.
Waterton & Dittmer [15]	Discusses how memorials employing sensory stimuli such as lighting, sound, and vibration can offer immersive experiences, allowing visitors to connect deeply with historical events, illustrated by the sensory design of the Australian War Memorial’s Bomber Command exhibit.

Movement

Moving through architectural spaces enhances people’s sense of physical presence and sensory engagement. This concept improves physical embodiment, connection with space, and three-dimensional perception [2,45–48]. Heidegger’s concept of Da-sein, or "being-there", emphasizes the embodied nature of human existence, with movement shaping personal experiences and spatiality [46]. Wineman & Peponis discuss how spatial form affects movement, visual contact, and engagement in spaces, identifying constrained to free movement patterns that affect visitors and exhibit co-awareness and interaction [49]. Choi examines how the intelligibility and integration of spatial design can inspire visitors to explore museums independently, increasing their immersion [50].

Tzortzi offers a framework for categorizing museum spaces based on visitor movement and interaction, defining spaces that facilitate dwelling, control movement, guide circulation, or offer navigational choices, aiding curators in designing engaging visitor experiences through spatial narrative [8]. Accordingly, Tzortzi concludes that four types of spaces affect museum spatial organization and visitor experience: circulation spaces, often in rings, which allow movement and provide one way back, facilitating museum flow; enclosed occupation spaces which invite visitors to stay and interact with exhibits, often acting as dead-ends; controlled spaces which manage visitor flow by providing a single entry and exit route to other spaces, including occupation spaces; choice spaces which give visitors more freedom to explore the museum by offering multiple routes. Table 7 summarizes these scholars’ findings on museum movement and how spatial design enhances visitor experience.

**Table 7:** The role of movement in shaping the sensory and spatial experience as highlighted by scholars.

Scholar	Key Contributions
**Choi** [50]	Examined the role of intelligibility and integration in spatial design, fostering individual visitor journeys through the museum.
**Heidegger et al.** [46]	Focus on the embodied and temporal nature of existence, with movement allowing dynamic engagement with the world, shaping the visitor’s subjective experience and sense of self.
**Wineman & Peponis** [49]	Assert that spatial form influences movement and engagement with exhibits, with patterns of movement ranging from highly constrained to entirely free, affecting the unfolding experience.
**Tzortzi** [8]	Developed a framework for classifying museum spaces based on visitor movement, with types of spaces ranging from occupation to choice spaces, impacting visitor interaction and experience.

Embracing the Void: Absence as a Narrative Tool in Museum Spaces

In museums, spatiality and absence shape visitor-exhibit interaction and awareness. Exhibit-free spaces can spark thought and add to the narrative. This idea of absence encourages visitors to explore the narrative, fostering discourse and interpretation among peers, experts, and storytellers, enhancing visitors’ engagement with history.

Space is used well to evoke memory and reflection at the Red Location Museum Memorial. Its “Memory Boxes”—large cubicles resembling migrant workers’ containers—reject traditional exhibition layouts in favor of equality and contemplation. These boxes, which represent different aspects of the workers’ lives, are interspersed with empty spaces to encourage reflection and emphasize how spatial gaps can enhance storytelling [51].

#### 2.2.2 Conscious experience (felt-body)

The felt body, or conscious experience, blends sensations, emotions, and thoughts to form inner experiences. This aspect of embodiment emphasizes how consciousness shapes environmental perception and comprehension. Culture influences how the felt body processes emotional and cognitive experiences. Narratives, motifs, and spatial designs reflect these cultural elements, enhancing understanding and emotional connection to the spaces around and illustrating the complex relationship between internal and external experiences.

*2*.*2*.*2*.*1 Emotional Experience and Cognitive Experience*. The depth of interaction with physical and sensory tools shapes visitors’ cognitive and emotional connections to museum narratives and themes. Cognitive experiences include perception and memory, tailored to individual knowledge and beliefs [52], while emotional experiences include stimuli-induced feelings [53].

Cognitive assessments of events affect emotional responses and vice versa. Emotions affect attention, memory, and decision-making by enhancing or distorting cognitive processes and memory retention [54–58].

The relationship between emotions and cognition, while complex, underscores the significance of strong emotional stimuli in shaping memory and judgments and in enriching visitor encounters [59–63].

Following Tzortzi, the embodied theory of meaning asserts the inseparability of cognition and emotion and emphasizes physical engagement in architectural spaces [8]. Architects, such as Zumthor, Pallasmaa, Roberts, and Witcomb, advocate for architecture that engages emotions and senses, creating an embodied and affective space. This holistic approach to architecture and museums emphasizes the importance of cognitive understanding and emotional resonance in visitor experience.

*2*.*2*.*2*.*2 Influential Factor*: *Culture*. Cultural attributes make narratives more understandable and impactful, according to Keunhye Lee [35]. Motifs, materials, and spatial arrangements convey cultural heritage stories and meanings through physical and sensory use. They help interpret the designer’s narrative by evoking culturally specific memories and emotions, connecting the abstract design concept to the occupants’ shared cultural experiences. Culture in architecture creates emotional connections and improves cognitive understanding of the narrative contained within a space.

**Fig 3:**
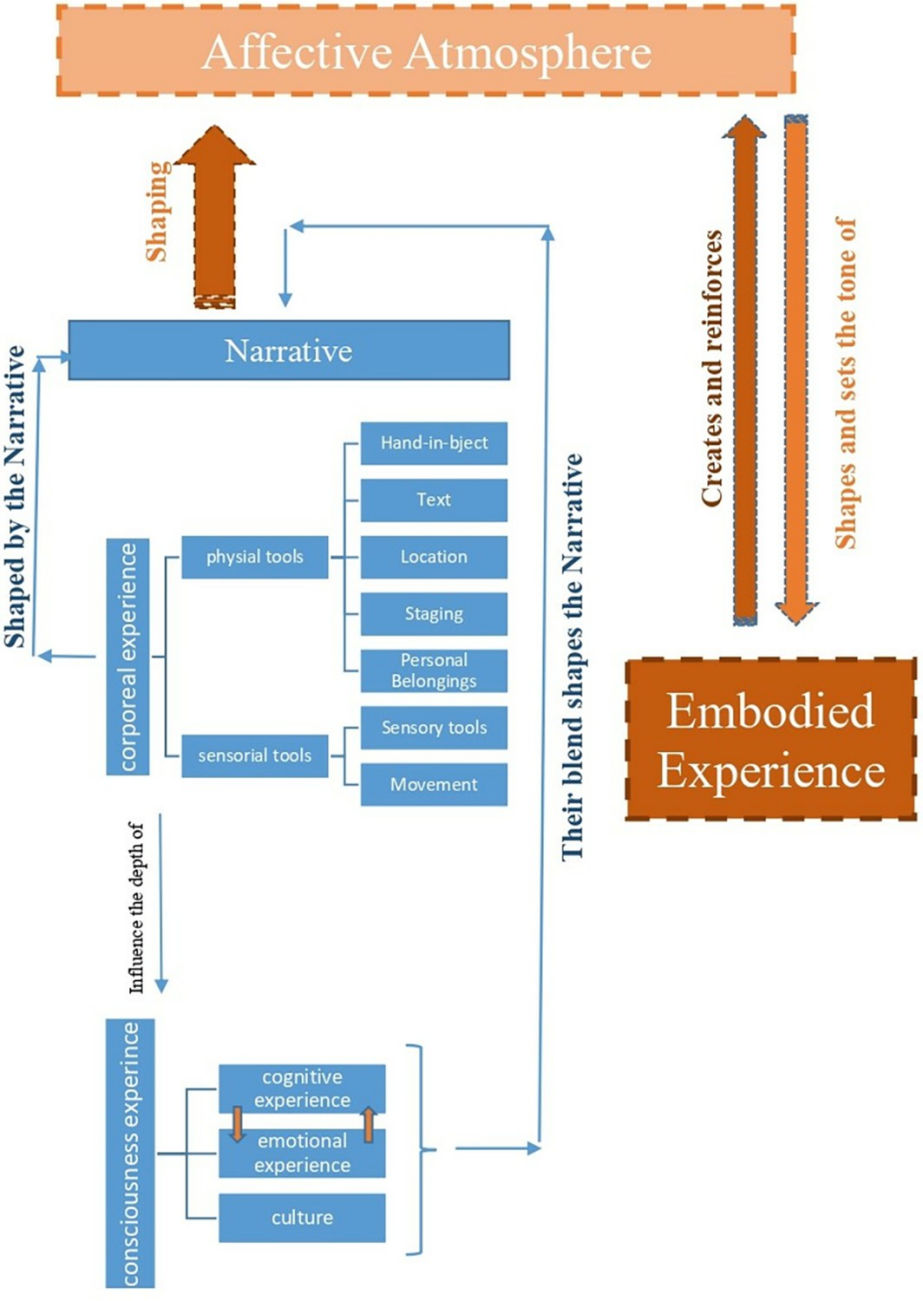
An overview of the literature exploring the inter-relationship between the parameters that shape the affective atmosphere.

The diagram in (Fig 3) summarizes the literature and shows how the parameters that shape the affective atmosphere interact to create an embodied experience.

## 3. Methodology

The literature review has explored the nature of embodied experience in museums, drawing on various international examples. Based on these theoretical and practical attributes, this research has developed a conceptual framework for exploring both the key concepts and the ways they are realized to create visitor experiences at RTM.

- The first step involves describing RTM episodes unfolding and their layout sequence.
- The second involves conducting an online survey independent of RTM to capture Jordanians’ vivid memories of the most memorable battles, characters, and moments in military history.
- In the third step, each episode’s composition is compared to the tools available for depicting these narratives and how intensely they may convey them.
- The fourth step involves tracking visitors’ engagement with the episodes and content to track behavioral patterns.
- The fifth step involves comparing the observed behaviors to visitors’ oral impressions in short structured interviews.
- The sixth step involves detailed questionnaires for the most and least engaged episodes.

**Fig 4:**
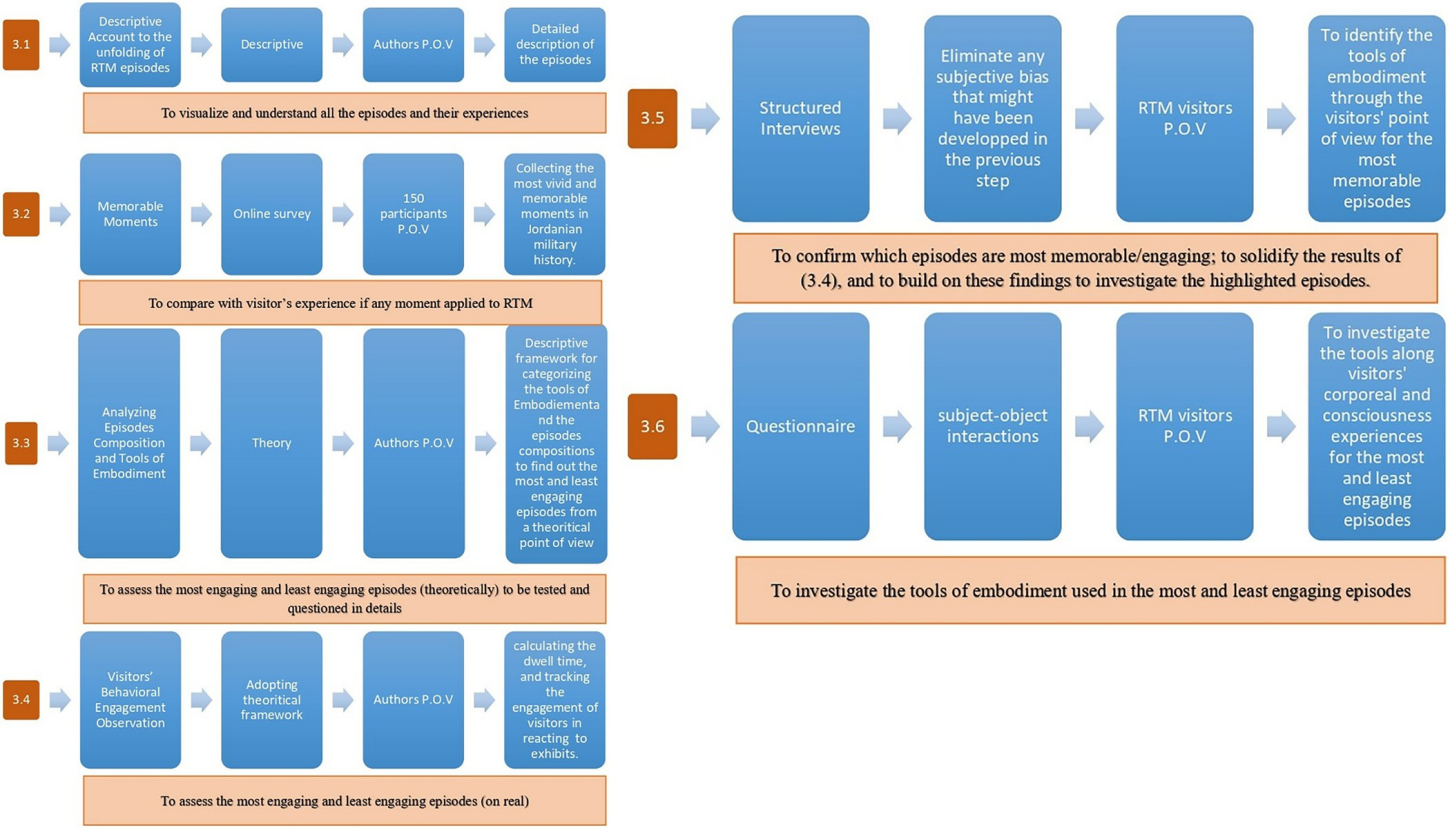
1. The methodological steps taken in this study from section 3.1 to 3.4. 2. The methodological steps taken in this study from section 3.5 to 3.6.

The methodology steps are summarized in Fig 4-1 and 4-2.

### 3.1 Descriptive account of unfolding RTM episodes

**Fig 5:**
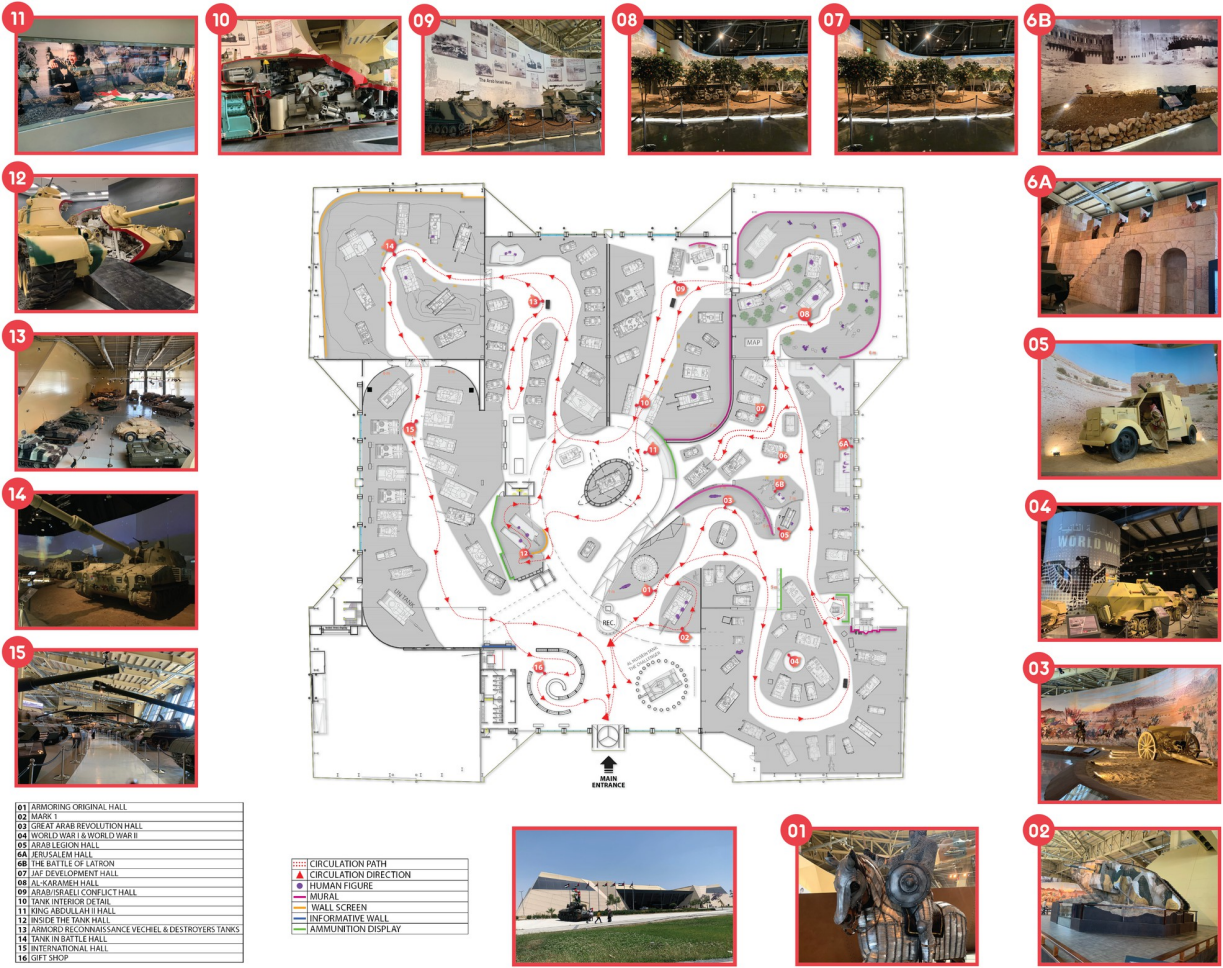
RTM’s episodes.

As mentioned, Jordan’s RTM is a unique military and history museum. The Amman museum displays tanks and armored vehicles that have shaped Jordanian and regional history. The museum has tanks and other vehicles from World War I (WWI) to the present. Fig 5 illustrates episodes explored in RTM.

In the Armoring Original Hall (Episode 1) of the Jordanian RTM, a medieval horseman and Leonardo da Vinci’s 1487 tank model show early tank concepts and fantasies. Visitors then see the Mark 1 Tank (Episode 2), the first combat tank designed to cross trenches and difficult terrain, as a symbol of WWI. The tank is cut to show soldiers inside and beneath it and create a war scene. Following a global chronology, a mural depicting the Great Arab Revolution (Episode 3) and the era’s armed vehicles showcase Jordan’s role.

The following spaces are dedicated to WWI and World War II (WWII) (Episode 4), presenting tanks aligned according to the alliances during these wars and narrating the stories in relation to the armed capabilities of the opposing military coalitions. The next space portrays Transjordan during the same period; it is known as the Arab Legion Hall (Episode 5).

The narrative then shifts to the conflicts between Arabs and Israel from 1948 through to the 2000s, starting in Jerusalem Hall (Episode 6A), which exposes visitors to the narrative of the city, including battles like Wadi Albab (Episode 6A) and Al-Latrun (Episode 6B). This space also talks about the Jordan Armed Forces (JAF) development hall. This is followed by Al-Karameh Hall (Episode 8), where the 1968 battle between Jordan and Israel is depicted, offering a phenomenological experience with sensory elements enhancing the narrative.

The Arab-Israeli Conflict Halls (Episode 9) emphasize the Arab world’s role in the conflict with Israel. After that, visitors pass through a tank, cut in half (Episode 10), then enter King Abdulla-II Hall (Episode 11), a huge atrium lit by a skylight that displays the King’s personal military items, including a powerful tank and a Cobra attack helicopter.

Visitors next encounter the Inside the Tank Hall (Episode 12) and Armored Reconnaissance and Tank Destroyers (Episode 13). Before arriving in the Tank in Battle Hall (Episode 14), which immerses visitors in war with visual and auditory effects. This section of the museum ends with a tent with wax soldiers planning an invasion or studying war tactics. Episode 15 ends with a stunning view of the tanks in the International Hall.

Visitors can exit the museum or visit the gift shop (Episode 16) on the ground floor after the tour. Additional areas like the upper floor coffee shop and gaming center are part of the journey but not yet open.

### 3.2 Memorable moments

Researchers wanted to offer another perspective on the most significant events in Jordan’s military history in addition to describing RTM episodes. Their goal was to promote collective memory of key figures and battles independent of RTM and its design. To do this, they created an online survey asking random participants to name the most memorable modern event, character, or battle in the region.

A total of 150 respondents completed the survey. The Battle of Al-Karameh and the 1948 Palestinian massacres, especially Al-Tantura and Deir Yassin, were most mentioned. Participants vividly remembered Jerusalem and Al-Aqsa Mosque. They also remembered King Abdullah II, Sharif Hussein Bin Ali, and Hussein.

Episode 8 of RTM features the Battle of Al-Karameh. The museum design does not explore the history of the 1948 Palestinian massacres’. Jerusalem appears only in Jerusalem Hall (Episode 6A) along with the Battle of Al-Latrun (6B). Episode 11 introduces visitors to King Abdullah’s belongings and related events. Sharif Hussein Bin Ali is represented in Episode 3, and King Hussein is represented through his speech and photos in Episode 8. These memorable episodes are analyzed as this research explores how visitors interact with RTM episodes.

### 3.3 Analyzing the composition of episodes and tools of embodiment

Within the framework of the literature review, this step aims to understand the ways by which the design of the RTM episodes unfolds with their potential to connect with visitors. Table 8 below creates a checklist to filter the episodes according to the research’s literature review. It tests each episode according to its physical tools (hands-on objects, text, location, staging and personal belongings), and its sensory tools (sensory stimuli and movement).

**Table 8:** Researchers’ checklist; filtering the narrative according to its abstract physical and sensory tools.

	*Physical Tools*					*Sensory tools*		
**EPISODE**	**Hands-on objects**	**Text** Tank info: * Story: ** Poster: ***	**Location**	**Staging** **Description**	**Personal belongings**	**Sensory stimuli**	**Movement**	**Spatial characteristics**
**1-Armoring Original Hall**	N/A	*	N/A	The knight and Davinchi war tool), models only, no scene	N/A	N/A	Uniformly sequenced, from one side,	Circulation-Pass through
**2-Mark 1**	N/A	*	N/A	A scene showing soldiers inside the tank going over soldiers inside a trench	N/A	N/A	Up, down and around Free movement	Occupation space
**3-Great Arab Revolution hall**	N/A	***Posters and images of a train and a bridge.	N/A	Mural with a model of an Arabian knight on a horse. The mural shows the confronting of two armies; the Turkish and the Arabs.	N/A	N/A	Uniformly sequenced from one side	Circulation-Pass through
**4- WW1** **& WW2**	N/A	*	N/A	Tanks and army cars. At the end there is a scene for a burned house. Some weapons, mural poster of the burned buildings. No wax figures	N/A	N/A	Uniformly sequenced	Circulation-Pass through
**5-Arab Legion Hall**	N/A	* ** the Arab army and its role in the Arab Israeli conflict. Posters of real photos	N/A	Scene from wax figures; Arab army standing over an army car	N/A	N/A	Uniformly sequenced with free choice	Choice space
**6A-Jerusalem Hall**	N/A	*	N/A	A wall with stairs going up. Some soldiers (wax figures) on the ground prepping their weapons. Other soldiers guarding the wall from back and front. They are looking at something that we cannot spot.	N/A	N/A	Uniformly sequenced and randomly sequenced. See from one side. Cannot go up the stairs	Choice space
**6B-The battle Al-Latrun**	N/A	*	N/A	Mural of a building. Wax figure (soldier) is hiding in a pit as guarding the building	N/A	N/A	Randomly sequenced. At the visitor’s back when approaching Jerusalem Hall.	Choice space
**7-JAF Development Hall**	N/A	* ** the Arabization of the Jordanian Army	N/A	Tanks. Mural with some text No scene	N/A	N/A	Entirely free to go and watch or skip	Choice space
**8-Al-Karamah Hall**	N/A	* ** Introduction about Al-Karamah battle on the entry. Poster (a piece from newspaper) at the exit.	N/A	Model of the battle terrain displayed through projector at the beginning of the journey. Orange trees filling the space as they are covering some tanks. You can smell the oranges. Soldiers (wax figures) hiding in different positions; in pits, and on tanks. A big mural covering a scene of fire and big war is taking place. Murals. Songs of victory and resistance songs. Speech of King Hussein, with some picture of him, and some news at the exit declaring the victory). Dimmed lights.	N/A	Olfactory Audio	Highly constrained, entry and exit. Move around. Visitor is encircled by the scene on the left and the right.	Circulation-Pass through
**9-Arab/Israeli Conflict Hall**	N/A	* ***Newspapers on the mural. Hard to read.	N/A	On the right some tanks and on the left big mural with headings of newspapers. Wax figure over the tanks.	N/A	N/A	Randomly sequenced.	Circulation-Pass through
**10-Tank Interior detail**	N/A	N/A	N/A	Sectioned tank, with two soldiers inside (wax figures)	N/A	Tactility	Highly constrained. No way but to go through	Circulation-Pass through
**11-King Abdullah II Hall**	N/A	*	N/A	Helicopter, tank, personal belonging of king Abdullah II	yes	N/A	Uniformly sequenced, with a choice to change direction.	Circulation-Pass through
**12-Inside the Tank Hall**	N/A	N/A	N/A	Sectioned tank, with two soldiers inside (wax figures). Weapons. Wax models wearing different army uniform	N/A	Tactility	Entirely free to enter. Once you enter, it is uniformly sequenced	Occupation space
**13-Armored Reconnaissance Vehicles & Destroyers Tanks**	N/A	* Nothing written about the tent. You cannot read the map in the tent	N/A	Tanks. tent with soldiers inside planning for war tactics, holding a map. There is a scene, but not understood.	N/A	N/A	Uniformly sequenced	Circulation-Pass through
**14-Tank in Battle Hall**	N/A	*	N/A	Lights, sound of bombing, murals of bombing tanks, soldiers on tanks	N/A	Audio	Highly constrained.	Circulation-Pass through
**15-International Hall**	N/A	*	N/A	Tanks represented in a ceremonial perspective. No wax figures	N/A	N/A	Highly constrained	Circulation-Pass through

The analysis reveals significant variation in the deployment of embodiment tools across the museum’s episodes. The use of tools also differs, with certain episodes relying solely on physical means and others integrating both physical and sensory elements. The most immersive environment is found in Al-Karameh Hall, distinguished by its comprehensive sensory engagement, including auditory and olfactory experiences, alongside an enveloping staging that fully engages the visitor. In contrast, the Arab/Israeli Conflict Hall, Jerusalem Hall, and the Battle of Al-Latrun rank lower in terms of engagement, due to a less dynamic blend of physical and sensory tools. For a better visualization, Table 9 below shows the episodes in descending order based on how comprehensive the experience is in terms of physical and sensory tools, highlighting the highest and the least immersive episodes.

**Table 9:** Episodes listed in descending order from those using most tools to those using least tools.

Episode No.	Episode’s Name	Description
**8**	**Al-Karameh Hall**	Highly immersive with sounds, lights, smell, detailed narrative text, tactile elements, and a structured path for a full sensory experience.
**14**	**Tank in Battle Hall**	Engages visitors with sound effects, visual elements, and a constrained path to enhance realism.
**10**	**Tank Interior Detail**	Incorporates tactile elements and a highly constrained path for focused engagement.
**12**	**Inside the Tank Hall**	Features tactile interaction with sectioned tanks and wax figures, providing a moderate physical experience.
**5**	**Arab Legion Hall**	Uses wax figures, signs, and posters, offering good visual and physical representation but lacks sensory stimuli.
**11**	**King Abdullah II Hall**	Includes personal belongings and a mix of physical tools but lacks sensory elements.
**2**	**Mark 1**	Allows free movement with visual displays and some informational signs but lacks sensory engagement.
**7**	**JAF Development Hall**	Offers free movement and visual elements but minimal sensory stimuli.
**4**	**WW1 & WW2**	Provides structured viewing with visual displays and signs but lacks sensory engagement.
**1**	**Armoring Original Hall**	Structured path with visual models but no sensory or interactive tools.
**3**	**Great Arab Revolution Hall**	Visual engagement with murals and models but no sensory stimuli.
**13**	**Armored Reconnaissance Vehicles & Destroyers Tanks**	Displays tanks with minimal narrative or sensory tools.
**9**	**Arab/Israeli Conflict Hall**	Poor readability of text and lack of sensory stimuli result in an incomplete experience.
**6A**	**Jerusalem Hall**	Minimal text and static displays without interactive or sensory elements.
**6B**	**The Battle of Al-Latrun**	Relies on a mural and wax figure with minimal text, offering the least comprehensive experience.

### 3.4 Observation of visitors’ behavioral engagement

To analyze visitors’ relationship within the design of RTM, and to determine the interaction level of the visitors with the content of each episode, the study adapted the method of Wineman & Peponis (49) for observing visitors. Their method involved tracking dwell time (the time spent in each episode) as an indicator of deeper connection and interaction with the episodes and body behavior for signs of engagement like leaning in, taking pictures, or reading. This functionally bias-free method can help identify episodes that didn’t capture visitors’ attention’.

The research proposed for this paper was granted ethical approval no. S/1a/4/2023 by the University of Petra’s Ethical Approval Committee, and RTM administration approved the observational study, ensuring visitors were informed and consented to it verbally in the front desk when they bought their tickets, prioritizing ethics and privacy. Observations were taken on May 17, 20, 21, 25, and 27 in 2023 to capture weekend and weekday behaviors. Each visitor group—family, couple, or individual—was tracked as a single unit, yielding 40 unique observations.

**Fig 6:**
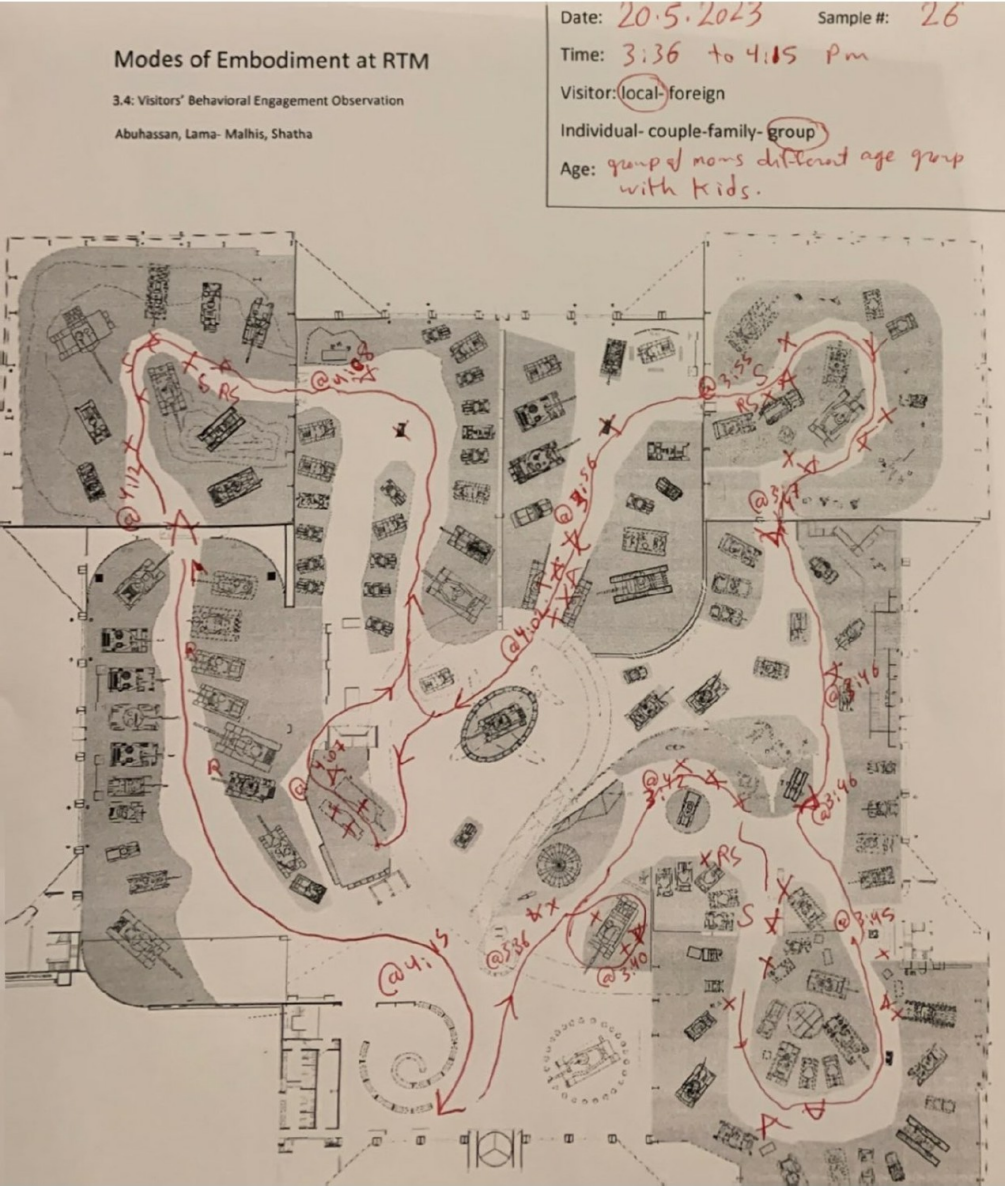
A sample of a tracking map observing visitors’ engagement and dwell time. Legend as following: Time: @ 00:00; Engagement: X; Taking Photo: <; Information panel reading: R; Touchscreen engagement: S; Information touch screen reading: RS.

The map below (Fig 6) represents a sample of how researchers marked visitors’ engagement and dwell time: "X" for direct engagement, ">" for picture-taking, "S" for touchscreen engagement, and "R" or "RS" for information panel reading. Among local visitors (24 families, 2 couples, 6 individuals and 3 groups), dwell times varied from a brief 15 minutes to a full hour, with an approximate average of 35 minutes for the visit. In contrast, foreign visitors, including (2 families, 4 couples, 3 individuals, and 6 groups), displayed dwell times ranging from 45 minutes to 1 hour and 30 minutes, with an approximate average of an hour.

In order to make the 40 observations readable according to dwell time, the observations were collected in Table 10.

**Table 10:** Average dwell time in minutes for 40 tracked visitor units.

*Episode*	*Average dwell time* (min)	
	**Locals**	**Foreign**
**1-Armoring Original Hall**	**1**	**4**
**2-Mark 1**	**2**	**5**
**3-Great Arab Revolution hall**	**2**	**2**
**4- WW1** **& WW2**	**4**	**10**
**5-Arab Legion Hall**	**2**	**3**
**6A-Jerusalem Hall**	**0.5**	**2**
**6B-The battle Al-Latrun**	**0.5**	**2**
**7-JAF Development Hall**	**0.5**	**2**
**8-Al-Karamah Hall**	**7**	**4**
**9-Arab/Israeli Conflict Hall**	**1**	**5**
**10-Tank Interior detail**	**1**	**1**
**11-King Abdullah II Hall**	**1**	**3**
**12-Inside the Tank Hall**	**1**	**2**
**13-Armored Reconnaissance Vehicles & Destroyer Tanks**	**2**	**5**
**14-Tank in Battle Hall**	**3**	**3**
**15-International Hall**	**2**	**8**
**Total**	**30.5**	**61**

**Fig 7:**
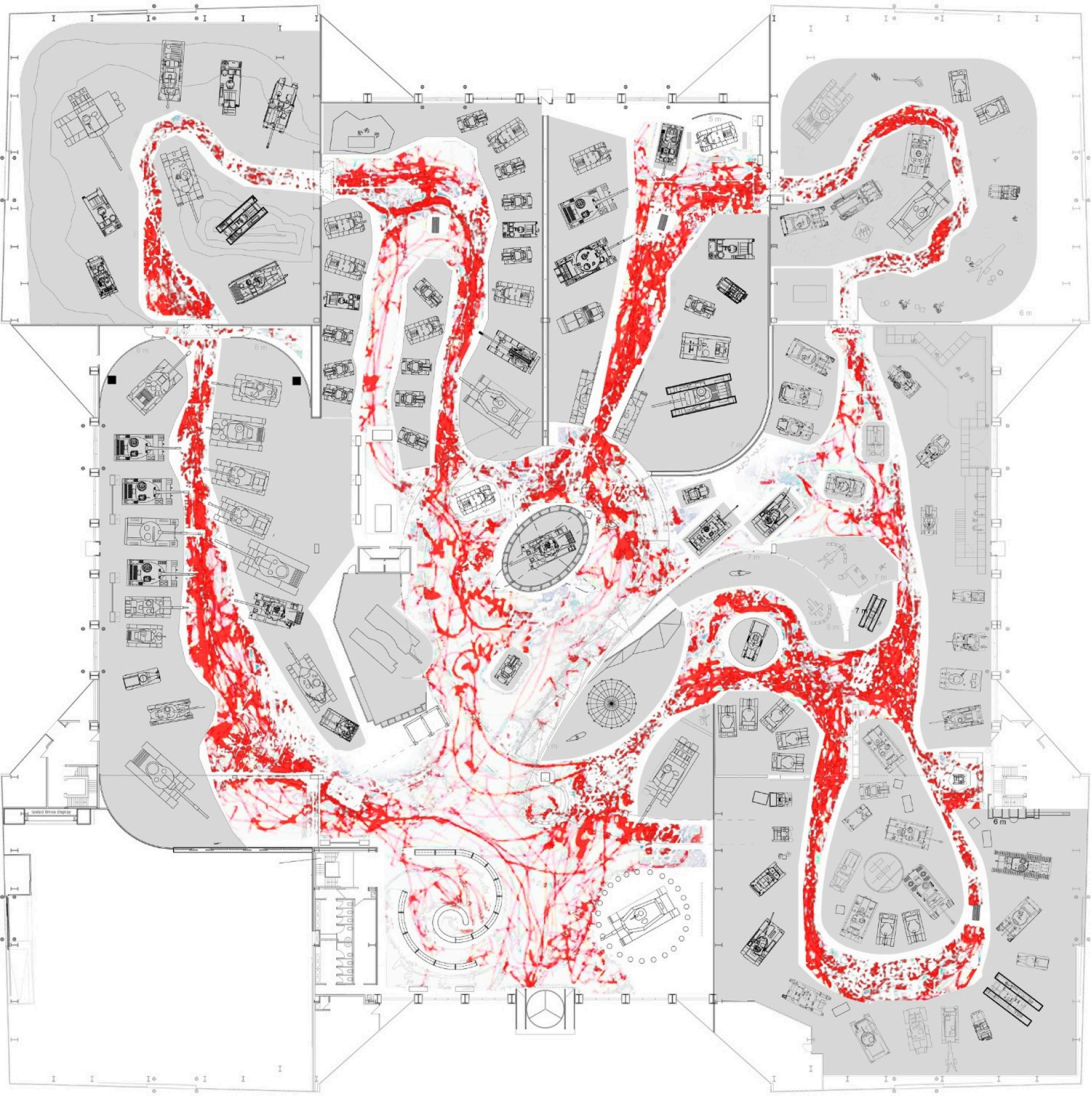
The overlapped tracking maps.

In order to understand the type of engagement, all tracking maps were overlapped to capture the most intense engagement pattern, as in the map below in Fig 7.

The overlapping of the tracking map shows that there was equal engagement in Episodes 1, 2, 3, 4 and 5 in taking pictures, and reading, then it faded significantly in Episodes 6A and 6B and 7 up to Episode 8. At Episode 8 the overlapped tracking map shows that there was a high level of engagement, the most for the whole map. Episodes 9, 11 and 13 were interacted with equally. Interactions with Episodes 10 and 14 interactions were significant especially with families with children. In Episode 10 the interaction was physical with the soldiers in the sectioned tank; Children touched the soldiers and took picture inside the tank. Episode 12 is designated as a private zone, so not all visitors went inside. The interaction was minimal. Visitors interacted a lot with Episode 15 due to the big number of tanks that are arranged in a ceremonial layout.

Visitors seemed to prefer taking photos in most episodes, as a kind of interaction, and were less interactive with reading than with other display tools like touch screens.

**Fig 8:**
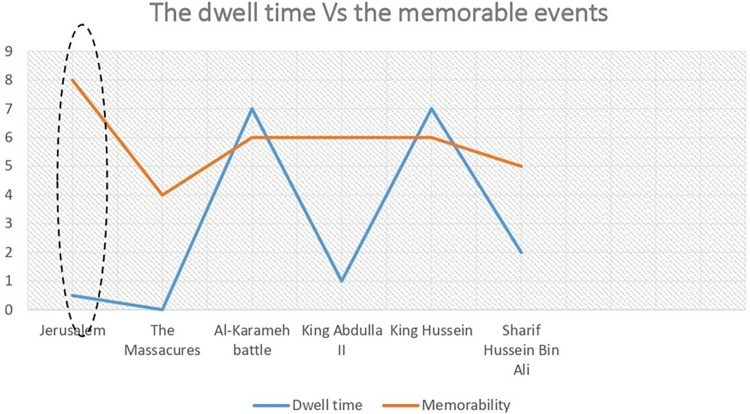
Dwell time vs memorable events for the main events recalled by locals.

When the findings of the engagement study were compared with those of the memorable moments survey in Section 3.2, a contrast emerged (Fig 8). Despite visitors’ vivid memories of Jerusalem, it did not receive high dwell time, as shown in Table 10. Although Jerusalem and the Arab-Israeli wars played an influential role in visitors’ memories, the museum’s design appears to reduce their engagement with these sections. This discrepancy leads the study to investigate why the Al-Karameh hall is highly engaging and has a long dwell time, while Jerusalem Hall and the Battle of Al-Latrun receive the least interaction and dwell time.

### 3.5 Structured interviews

To enrich the findings and eliminate any bias from the visitor observation phase, structured interviews were conducted with 40 randomly selected visitors at RTM, following ethical approval no. S/1a/4/2023 obtained from University of Petra’s ethical approval committee. Participants were verbally informed, and this was witnessed under the administration of RTM. The interviews aimed to gather feedback about visitors’ experiences at the museum after completing their tours, noting that only 10 interviewees had previously visited the museum. The questions included:

What is the most memorable episode and why?

How do you know the story of the episode you most remembered? From memory or from reading the text?

Defining the tools of embodiment (from interviewees’ memory)

3.1-Have you read signs in the museum? Where?

3.2-In which episode(s), did the scene (staging) attract your attention?

3.3-Where in the museum were your senses aroused?

What would you like to see more of in the future?

While Table 8 assessed the modes of embodiment at RTM from the researchers’ literature review perspective, the following table, Table 11, collects the feedback from visitors’ personal experiences.

**Table 11:** Visitors’ structured interview answers, where the numbers below show the number of interviewees who agreed on the same point.

Episode no. and Name	Q1: Most memorable episode	Reason	Q2: Narrativity		Q3: Physical and Sensory tools recognized		
			Storytelling by memory	Storytelling by text	3.1 Text Tanks info	3.2 Staging	3.3 Sensory stimuli
**1-Armoring Original Hall**				1		7	
**2-Mark 1**				5	6	14	2
**3-Great Arab Revolution hall**	2	They know the story	1		4	6	
**W4- W1 & WW2**			4		5	14	1
**5-Arab Legion Hall**					7	2	
**6A-Jerusalem Hall**			3			5	1
**6B-The battle of Al-Latrun**						no	
**7-JAF Development Hall**						3	
**8-Al-Karameh Hall**	21	Trees, the King’s speech, soldiers	13	10	9	10	15
**9-Arab/Israeli Conflict Hall**				5	1	7	1
**10-Tank Interior detail**				2			
**11-King Abdullah II Hlall**				1		8	3
**12-Inside the Tank Hall**							
**13-Armored Reconnaissance Vehicles & Destroyers Tanks**				1	8	8	2
**14-Tank in Battle Hall**	12	The war sound and bombs		7		8	14
**15-International Hall**				5	5	14	5
**Most engaging exhibits**							
**Sectioned tanks- Episodes 10 and 12, Vagans Arab Army Car- Episode 5, T series tanks- Episodes 13, Mark I- Episode 2**							
**What would you like to see more in the future?**							
**More weapons, Movies, VR, Touching the tanks and climbing on, Take care of Great Arab Revolution hall, Prayer room.**							

The interviewees were allowed to agree on more than one episode, or none; Hands-on objects, locations, and personal belongings were not recorded as they were deemed inapplicable according to the researchers’ checklist. Movement was also excluded from the checklist, as it occurs subconsciously, influenced by either mimicking other visitors’ movements, personal choice or design layout control.

The following paragraph summarizes the interviewees responses to the selected tools that were examined in the structured interviews.

- Visitors at RTM rated Al-Karameh Hall as the most memorable, especially for families with children due to its interactive visual and audio effects. The analysis examined story comprehension, sensory engagement, and staging.
- Al-Karameh Hall scored highest in terms of the clarity of the narrative and storytelling, followed by the Armored Reconnaissance Vehicles & Destroyers Tanks (Episode 13). Mark 1 (Episode 2) and the Arab Legion Hall (Episode 5) had mixed storytelling success, while the Battle of Al-Latrun was particularly weak.
- Al-Karameh Hall again led in terms of sensory engagement with a rich interactive experience, followed by Episode 13. The Battle of Al-Latrun was less effective than the Tank in Battle Hall, indicating varied sensory stimulation across exhibits.
- Al-Karameh Hall significantly improved visitor experience, while Mark 1 and Tank in Battle Hall performed well but not as well as Al-Karameh Hall. The Great Arab Revolution Hall scored moderately, while the Battle of Al-Latrun and Tank Interior Detail were unremarkable.
- Table 9 lists Al-Karameh Hall as the most engaging episode, while Jerusalem Hall and the Battle of Al-Latrun as less interactive. Embodiment modes in Al-Karameh Hall and their effects on cognition and emotion need further study. We need to investigate why Jerusalem and the Battle of Al-Latrun were less engaging.

### 3.6 Questionnaire

Since the first level observational method and structural interviews identified Al-Karameh as the episode most interacted with in the museum and Jerusalem Al-Latrun as the least, it is time to zoom in on those episodes and investigate the tools used and their impact on visitor experience.

The RTM questionnaire and the consent was waived and approved by the University of Petra’s Ethical Committee (no. S/1a/4/2023). Participants were informed about the consent in writing. On the first page of the questionnaire, it states:

*Dear Participant;*

*We thank you for your interest and willingness to participate in filling out the questionnaire regarding the study of visualization methods in the Royal Tank Museum*. *This study aims to assess the level of visitor interaction with the exhibits in the museum in order to identify elements of interaction and work on proposing any future improvements*. *Your name will not be associated with the answers*, *and your responses will be used solely for research purposes*. *Filling out this questionnaire signifies your consent to participate*”.

Responses from minors were collected through their guardians, who completed the form on behalf of their children.

It was carefully designed around five main themes: narrative, physical experience, sensory experience, emotional experience, and immersion. These themes help explain how narratives affect visitors physically, sensually, emotionally and cognitively. These themes also define whether the experience is embodied. The analysis now focuses on the dual relationship between the visitor and the tools. Visitors are considered active participants who share their personal experiences.

After demographic questions like age, gender, and nationality, the questionnaire moved on to more detailed questions in three sections. The first section covered Al-Karameh, with 25 questions on the following themes: 4 on narrative, 8 on physical tools, 4 on sensory tools, 6 on emotional experience, and 2 on immersion. The second section, Jerusalem Hall, had 11 questions: 4 on narrative, 2 on physical tools, 2 on sensory-tools, 2 on emotional experience, and 1 on immersion. The third section, on Al-Latrun Battle, had 9 questions: 3 on narrative, 3 on physical tools, 2 on emotional experience, and 1 on immersion.

Each section of these thematic areas includes open-ended, multiple-choice, and sketching questions. The word choice in open-ended questions helped determine whether responses were emotional or cognitive. Multiple-choice questions helped distinguish emotional and cognitive dimensions, while sketching exercises emphasized cognitive reasoning.

The study included 78 participants, with 70 completed questionnaires. Conducted at RTM, the questionnaire was given to visitors once they had completed their visit. Participants were randomly selected as they finished their tour. The data collection occurred from June to August 2023, encompassing midweek and weekend times. Among the participants, 90% were Jordanians, and 10% were non-Jordanians. The gender distribution was 45.1% female and 45.9% male. The age distribution was as follows: 13 to 17 years (16.2%), 18 to 35 years (56.8%), 36 to 55 years (23.0%), and 56 to 70 years (4.1%). The detailed questionnaire for the three episodes which were investigated with their results can be found in S1 Appendix.

To help with navigation of S1 Appendix, Table 12 presents a sample of how each question was designed, read by the participant, and interpreted by the researchers. For example, Question 4, relating to Al-Karameh Hall was crafted around the theme of: the physical tool of the corporeal experience and under the sub theme of (text). The nature of this question measured the conscious experience of the participant at the cognitive level. Question 16 on the other hand was crafted around the theme of the emotional experience of the participant as part of the conscious experience.

**Table 12:** A sample of the questions addressing the five themes for Al-Karameh episode along with the analysis of the 70 participants’ responses.

Main thematic questions	Sub thematic questions	Questions	Cognitive /Emotional	Result of the answers of 70 participants
**Episode 8: Al-karameh Hall**				
**A: Narrative**		Q1-In your own words, can you please summarize the story of the Battle of Al-Karameh. If you don’t know it please write:”I don’t know”	Cognitive	Most of the participants could summarize the story of the Battle of Al-Karameh. (n = 70, 94.6%), about two third of them knew the real story of the Battle of Al-Karameh which is not far from the content of the story.
**B: Physical**	**B.1 Text**	Q4-Have you read the sign that exists at the beginning of Al-Karameh Hall? Yes No	Cognitive	In terms of museum signs, over two-thirds of participants (71.6%) reported reading the signs at the entrance of Al-Karameh Hall, while 77.0% read the signs and information on the tanks at Al-Karameh Hall.
**B: Physical**	**B.2: Staging**	Q7-What is the element that was responsible for narrating the story at the Karameh Hall for you? You may choose more than one answer the tanks the soldiers the orange tress the murals The explosion sound and fire The songs Nothing was responsible and I did not get the story	Cognitive	Based on Fig 2, most participants indicated that tanks, followed by soldiers and orange trees, were the key elements in the museum responsible for conveying the story in the Karameh Hall. Songs were identified as the least prominent element in the museum for narrating the story at the Karameh Hall.
**C: Sensorial**	**C1: senses**	Q9-Did you feel the heat of the battle during your visit to the Karameh Hall?	Emotional	More than half of the participants said they felt the heat of the battle during their visit to the Karameh Hall (43 ’yes’ 31 ’no’),
**C: Sensorial**	**C.2: Movement**	Q11-On the map below, kindly assign: if there is/are a specific position from which you observed the whole scene of the battle, kindly assign it with “X on the map. If there is no specific point, please write” No” kindly assign your stops at Al Karameh Hall through using this symbol on the map: Is there a favorite tank at this section? Please encircle it, or else, write:” No”	Cognitive	11 of 70 did not answer this question 7 of 67 couldn’t define a spot to see the whole story 8 of 67 couldn’t define a specific tank they recall Most spots focused on the entrance spot, middle spot and exit spot to see the whole story. In the same distribution they also remember their stops. The most special tank was on the right front of the entrance
**D: Emotional Experience**		Q16-Can you please assign the above feeling if it hits your body on this graph? you may use X or circles to draw.	Emotional	13 out 70 did not answer this Others answered Most answers focused on the heart and mind As if it is emotional and cognitive attached. Emotional was more.
**D: Emotional Experience**		Q19- How did How didKing Hussein’s speech made you feel Pride and happiness Anger and sadness Nothing	Emotional	The figure above shows that the majority of the participants felt pride and happiness during listening to King Hussein’s speech in Al-Karameh Hall.
**E: Immersion**		Q27-Did you feel as if you were in the battlefield or one of the soldiers while visiting the Karama Hall? Yes -No	Emotional	63.5% believe they are on the battlefield or one of the soldiers while visiting the Karameh Hall.

## 4.Discussion

### 4.1 Al-Karameh Hall: Episode 8

#### 4.1.1 Narrative as a tool

The narrative in Al-Karameh Hall is effective. A significant 94.6% of participants could understand and recount the Battle of Al-Karameh in their own words, with 78.6% understanding the sequence of events. This shows that the narrative design engaged most visitors, familiarizing them with the battle. Tanks, soldiers, and orange trees were essential in telling the story, but songs were less effective.

#### 4.1.2 Text

Text is crucial in this episode. Over two-thirds (71.6%) of participants read the text, which helped them understand the exhibit and bridged the gap between visual displays and the battle narrative. This integration helped visitors understand the events without prior knowledge.

#### 4.1.3 Staging

Episode 8 storytelling depends on staging. Tanks, soldiers, and orange trees helped make the battle engaging. The orange trees gave the exhibit legitimacy, nationality, and emotion. They made the battle depiction more realistic, enhancing the exhibit’s historical context and emotional impact. Military scenes and murals depicted pride and soldiers’ stories, capturing the emotional impact of the battle. Music enhanced the emotional atmosphere and overall ambiance, as recognized by 55.4% of participants, but it was less impactful than the visual and physical elements.

#### 4.1.4 Sensorial impact

Episode 8’s sensory elements enhanced visitor immersion. More than half of the participants (43 out of 70) felt the battle heat during their visit. Physical sensations made the scene more realistic and tangible, helping visitors connect emotionally with the historical event.

The episode’s layout and movement let visitors see the scene from different angles, improving their understanding and connection to the story. Episode 8’s limited entry and exit encircled visitors creating a controlled movement that enhanced the immersive experience.

Visitor emotions were greatly affected by songs and speeches. Seventy-seven percent said the songs brought them back to the victory and feelings of pride and patriotism, and the same percentage said King Hussein’s speech did. These sensory elements made the exhibit more realistic and emotional, making it powerful and memorable.

#### 4.1.5 Emotional impact

Episode 8 is emotionally powerful. The exhibit connected visitors to the soldiers, with 83.8% reporting glory and pride, and a sense that they were emotionally and cognitively engaged.

The exhibit’s realistic and immersive nature was shown by the fact that 60.8% of visitors felt the war’s difficulty and heat while walking among the tanks.

Seventy-seven percent felt pride and happiness after hearing King Hussein’s speech. This exhibit element enhanced the emotional connection to historical events and King Hussein’s leadership.

Most participants focused on the heart and mind when asked to assign their feelings to a body part, indicating that Al-Karameh Hall excels at cognitive and deep emotional engagement, making it a powerful and memorable historical exhibit.

#### 4.1.6 Cognitive vs emotional

The exhibit’s cognitive aspects emphasize historical accuracy and logical sequence. The cognitive elements of the exhibit successfully convey historical information to visitors, as shown by the high percentage of participants who could summarize the story (94.6%), re-tell events correctly (78.5%), and two-thirds accurately reflecting the content.

However, sensory and immersive elements evoke pride, empathy, and connection. Physical, auditory, and realistic staging create a powerful emotional experience that makes history more real.

#### 4.1.7 Affective atmosphere and embodiment

During the Al-Karameh episode, 63.5% of visitors felt like they were on the battlefield or one of the soldiers. Physical and sensorial tools integrated the Al-Karameh Hall narrative into corporeal experience. The battlefield felt more real because of the physical (tanks, soldiers, orange trees, mural) and sensorial (heat, sound, song, king’s speech, and movement) tools.

Cognitive experience formed due to the fact that visitors could understand historical facts and the logical sequence of events and that they were transmitted accurately. The emotional experience of the King’s speech and patriotic songs contributed to a conscious experience.

The effective use of narrative and the blend of corporeal and conscious experiences through physical, sensorial, cognitive, and emotional tools, in addition to prior knowledge of the battle and its cultural influence, creates a realistic and engaging environment that fosters an affective atmosphere and a deep sense of embodiment among visitors.

### 4.2 Jerusalem Hall: Episode 6A

#### 4.2.1 Narrative

Episode 6A’s narrative impact depends on visitors’ historical context recognition and understanding. The data indicate that many participants struggled with this. Only 18 of 70 participants correctly identified the exhibit and battle. Those who correctly identified the battle had prior knowledge. This reliance on prior knowledge suggests the exhibit may struggle to educate visitors unfamiliar with history. This suggests Episode 6A’s narrative elements may not be well-communicated.

Overall, 43.2% of participants correctly described the scene as a war scene with soldiers defending their territory without mentioning a battle or location. Visitors understood the battle field context but not the historical context. Lack of narrative cues may explain this partial comprehension.

#### 4.2.2 Staging

Episode 6A’s design and aesthetic received a rating of 3.95 out of 5, indicating that participants thought it was somewhat excellent. This rating indicates that the scene’s visual arrangement and artistic execution made it engaging and visually appealing. Participants understood the context thanks to the wall, soldiers, and tank. Fifty percent of participants stated the wall helped them understand the defensive barrier scene. However, due to narrative ambiguity, many visitors didn’t understand the scene despite its aesthetic appeal.

#### 4.2.3 Sensorial impact

The sensorial impact in Episode 6A was significantly attributed to visitors’ curiosity and desire to interact with the exhibit. A notable 87.8% of visitors wanted to climb the stairs to see what the soldiers were seeing. This suggests that the staging stimulates visitors’ senses and encourages them to explore.

However, understanding the exhibit holistically failed because participants who stopped to view the wall in Episode 6A often missed Al-Latrun Battle because it was behind them. Due to the exhibit’s layout and flow, visitors missed one of the main parts in the story of Jerusalem, leading to a fragmented understanding.

#### 4.2.4 Emotional impact

Episode 6A had positive emotional engagement, but responses varied. The majority (64.9%) felt victory and empathy due to the abstract representation of the wall as a defensive barrier, but they did not realize that this victory was the patriotic Bab Al-Wad battle at Jerusalem’s walls. Moreover, many visitors had no strong emotional reaction. A lack of clarity in the narrative, weak prior knowledge among visitors, and the display’s disconnection from the Battle of Al-Latrun in terms of layout can explain these diverse feelings and varied comprehension.

Cognitive vs. Emotional Experience:The cognitive aspects of Episode 6A show that while some visitors understood key narrative elements, many struggled to understand the historical context. Content and interpretive materials need to be improved due to the current reliance on prior knowledge and the difficulty of connecting scenes to the narrative. The exhibit was emotionally successful, but historical facts, narratives, and holistic context did not evoke the emotions.

#### 4.2.5 Affective atmosphere and embodiment

Overall, 80% of participants said they didn’t feel part of the scene. The gap between visual appreciation and emotional or physical immersion was significant.

Jerusalem Hall’s narrative impact depends on visitors’ historical knowledge. Although the wall, soldiers, and tanks helped participants understand a battle scene, the staging did not tell the full story, which hindered their understanding. The episode’s fragmented layout hindered a holistic understanding of Jerusalem Hall, resulting in a negative corporeal experience that failed to evoke a conscious experience.

The historical context was difficult for many visitors to grasp. Due to a lack of historical context and facts, the episode was emotionally engaging but insufficient.

Despite the high staging potential, the lack of narrative clarity and interactive elements prevented visitors from fully immersing themselves in the historical context to create a strong sense of presence. This lack of an affective atmosphere prevented visitors from having an embodied experience.

### 4.3 Al-Latrun Battle: Episode 6B

#### 4.3.1 Narrative

Episode 6B which focuses on the Battle of Al-Latrun, evoked mixed reaction in relation to the narrative effects depending on visitors’ ability to recognize the area and its historical significance. Despite 67.6% of visitors recognizing the area, two-thirds did not know the name of the battle, indicating a disconnect between the visual presentation and historical narrative. Better storytelling tools are needed due to this gap in understanding.

#### 4.3.2 Staging

Despite 71.6% rating Episode 6B perfect and complete, many participants did not understand the story.

#### 4.3.3 Sensorial impact

The failure of respondents to recognize the relationship between Sections 6A and 6B is a major issue. Only 33.8% knew both sections were about Jerusalem. The lack of recognition of Episodes 6A and 6B’s relationship also hindered comprehension. Visitors were not given enough context about Jerusalem and the Al-Latrun Battle by the layout and staging.

#### 4.4.4 Emotional impact

Visitor reactions to Episode 6B were mostly neutral. The had moderate empathy for the soldiers which demonstrated a personal connection but not enough to cause strong emotions. The lack of a clear narrative, visually appealing but emotionally insufficient staging, and a disconnect between Episodes (6A) and (6B) limited narrative continuity and emotional engagement.

#### 4.4.5 Cognitive vs. emotional impact

Visitors understood staging elements but struggled with the historical context. The exhibit evoked mixed emotions. The moderate empathy visitors felt for soldiers contrasted with a high percentage of participants feeling nothing. This suggests that the exhibit has the potential to emotionally engage visitors, but it does so inconsistently.

#### 4.4.6 Affective atmosphere and embodiment

Soldiers, murals, and cannons created a realistic, engaging scene and connected visitors to the scene, but only half felt as if they were part of the battle.

The visual presentation and historical narrative were inconsistent. The soldier staging, canon, and mural were the only elements present which helped visitors understand Jerusalem and the Al-Latrun Battle. The lack of recognition of Episodes 6A and 6B’s relationship weakened the sensorial tool and diluted the corporeal experience.

Staging elements helped the exhibit connect with visitors cognitively, but the historical context was lacking. Since this area failed to engage visitors emotionally engage, the conscious experience was negatively impacted.

**Fig 9:**
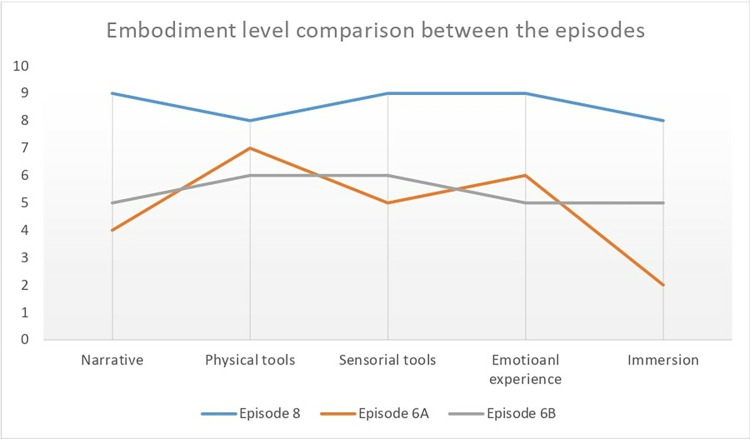
Comparison of embodiment levels for Episodes 8, 6A and 6B.

Jerusalem Hall (Episodes 6A and 6B) lacked a clear narrative, was disconnected between sections, and offered poor emotional and cognitive engagement, making it difficult to create an affective atmosphere or embodied experience. See Fig 9.

According to the discussion above and the chart below in Fig 9, Al-Karameh Hall excels in both cognitive and emotional dimensions to create an embodied experience. Episodes (6A) and (6B) in Jerusalem Hall demonstrate that while the staging and visual elements are effective, they need to be complemented with clear storytelling and interactive features to enhance visitors’ cognitive and emotional engagement and a deeper embodied experience.

## 5.Conclusion

Through its combined documentary and practical-theoretical interpretive approach, this paper contributes to the literature by documenting RTM’s attributes and the experiences it offers to visitors. It bridges the physicality of the form to explore the nature of the embodied experience it offers.

The research established a theoretical framework for describing the embodied experience offered by museums and RTM. It explained the theoretical foundations of embodied experiences and how narrative tools create affective atmospheres. The study developed a theoretical model that transforms corporeality and consciousness into tools that can capture and register layers of intangible embodied experiences by examining how narratives manifest as tools. A key theoretical-practical achievement of this study is its ability to transcend traditional narrative forms and provide a clear framework for understanding, researching, and using narrative tools in museums.

The research clarifies RTM’s episode-setting approach and evaluates episodes from multiple perspectives to determine how tools affect visitors’ experiences. The study examines visitor engagement with RTM episodes and how corporeal and consciousness tools impact on the museum’s affective atmosphere and embodiment. It also layered these tools to understand how they created the museum’s atmosphere and visitor engagement. RTM’s fifteen episodes were thoroughly investigated, but many failed to engage visitors. Jerusalem Hall and the Battle of Al-Latrun were the least engaging, while Al-Karameh Hall ranked as the most engaging.

The emotionally charged and memorable Al-Karameh Hall showed the importance of a cohesive narrative supported by sensory and physical tools. Al-Karameh Hall’s staging creates an immersive and educational experience for visitors. It emphasizes a balanced approach that incorporates sound, smell, heat, and strategic movement. Episode 8 captures viewers with its tightly constrained entry and exit, creating an immersive experience. This controlled movement immerses visitors in sensory and visual elements, deepening their emotional and cognitive engagement with the historical narrative. Text was crucial for conveying detailed information and improving the visitor experience. This multisensory approach created an embodied experience.

Jerusalem Hall had mixed narrative effectiveness. Due to narrative ambiguity, many visitors were unable to understand the scene despite its aesthetic appeal. The wall, soldiers, and tank were visually pleasing. However, they did not provide enough context for visitors to understand the scene’s historical significance. Episode 6B on Al-Latrun Battle mirrors Episode 6A. Despite having high aesthetic ratings and visually compelling and educational elements (soldiers, murals, and cannons), the episode lacks narrative clarity and connectivity. This shows that despite the strong visuals in both episodes, its absence of a clear narrative hindered visitors’ from comprehensively understanding the exhibits, which affected their emotional experiences and meant that they failed to connect with historical events.

The clustered and fragmented layout of Episodes 6A and 6B made it difficult for participants to recognize that they were related and part of the same important zone (Jerusalem). Most people who stopped to look at the wall in Episode 6A missed Episode 6B because it was behind them. A fragmented understanding of Jerusalem resulted.

RTM captured corporeal modes in Episodes 6A and 6B’s aesthetic staging with physical tools. However, the lack of a narrative meant that visitors found it difficult to understand Jerusalem’s context and the significance of Al-Latrun Battle, which weakened their conscious experience (cognitive and emotional), diluted their role, and created a non-affective atmosphere, resulting in an unembodied experience.

Despite positive feedback from RTM’s visitors and by linking this research to theoretical discussions, there are ways to engage all visitors more consistently.

- Modes of movement and encounter.

Al-Karameh Hall has a one-way entrance-exit pattern. If the movement is forked at a certain point to force visitors to turn around, this would allow them see the entire mural and feel like they are in a battle rather than watching it. This encounter can be enhanced by increasing the level of tactility in the episode, for example by allowing visitors to climb into a tank or move around it.

- Spatial experience

Tank spaces and display methods greatly affect spatial experience. Some episodes feel contained because they’re in enclosed spaces, but others are linear. The spatial experience makes or breaks the experience. The International Hall’s tank-lined axial space immerses visitors, while Jerusalem and Al-Latrun Halls’ castle wall imagery, with shallow enclosures and fragmented settings, lacks a sense of completeness.

- Text design

Text design and content enhance the narrative. RTM text was technical and emotionless. Focusing on font size, quality, strategic placement in relation to the exhibit, narrated content, and seamless integration into the display could help the text to fit in better with the context and nature of the exhibits. This would improve the visitor’s cognitive and emotional experience, which influences consciousness and embodiment. In Episode 9 on the Arab-Israel wars, the text was unreadable, indicating that visitors were unlikely to be able to engage.

- Interactive sensorial impacts

Interactive elements, such as audio guides or visual markers, in addition to tactile elements or multisensory experiences, could direct visitors’ attention to key areas, enhance engagement, convey the historical significance of the scene, and ensure that no important parts of the narrative are missed.

- Personal belongings

Personal belongings in episodes can boost empathy and emotional depth, creating a more embodied experience. These could include martyrs’ clothes, weapons, and diaries to help visitors connect more with the exhibits.

The findings showed that RTM needs more diverse and immersive elements to boost the emotional resonance of exhibits and make it more universally engaging.

## Supporting information

S1 Appendix The questionnaire. (PDF)
