# Supplementary material for: Modes of embodiment for an immersed experience in museums: The Royal Tank Museum
Source: PLoS One. 2025 Jan 29;20(1):e0317402. doi: 10.1371/journal.pone.0317402 (PMC11778790; doi:10.1371/journal.pone.0317402)
Supplement: S1 Appendix — (PDF) [file pone.0317402.s001.pdf]

Appendix A

Main thematic questions	Sub thematic questions	Questions	Cognitive /Emotional	Result of the answers of 70 participants.
Episode 8: Al-karamah episode				
A:Narrative		1-In your own words, can you please summarize the story of the Battle of Al-Karamah. If you don't know it please write: "I don't know"	Cognitive	Most of the participants could summarize the story of the Battle of Al-Karamah. (n=70 ,94.6%), about two third of them know the real story of the Battle of Al-Karamah which is not Far from the content of the story.
A:Narrative		2-How do you know the story? You can choose more than one answer.<br><br>I already know it<br><br>I have read the sign at the beginning of this section<br><br>The guide of the museum told me the story<br><br>Through other means	Cognitive	As shown in Fig. 1, more than half of the participants had prior knowledge of the story of the Battle of Al-Karamah (62.1%), 28.2% from museum signs, 17.6% from other other means, and only 9.5% from the museum guide.<br>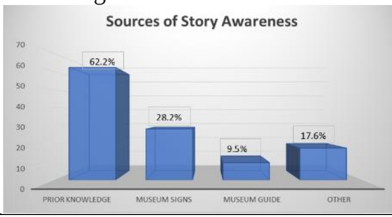
A:Narrative		3-Kindly re-arrange the following events according to what you have seen at Al-Karamah Episode<br>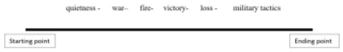<br><br>*<br>*The authors clarified the stages of the war along the timeline to ensure that participants understood the details and final result of the battle.	Cognitive	We counted who finished the answer with victory: to confirm they know or understand the story<br>55 out of 70 said victory
B: Physical	B.1 Text	4-Have you read the sign that exists at the beginning of Al-Karamah episode?<br>Yes<br>No	Cognitive	In terms of museum signs, over two-thirds of participants (71.6%) reporte d reading the signs at the entrance of Al-Karamah Episode, while 77.0% read the signs and information on the tank s at Al-Karamah Episode.
B: Physical	B.1 Text	5-have you read the signs and information of the tanks that exist at Al-Karamah episode?<br>Yes<br>No	Cognitive	In terms of the signs in the Al-Karamah Episode, around half of the participants (44.6%) chose some tanks to read about and read the signs at the entering point (45.9%), 29.7% read each sign belonging to any tank, 10.8% read the signs at the exit point, and 10.8% did not read anything.
A:Narrative		6-Do you think you need extra tools and methods to understand the story of Al-karamah battle? If yes, please suggest some	Cognitive	On the other hand, the participants reported that they needed extra tools and methods to understand the story of the Al-karamah battle such as more signs in the museum, videos that

				describe the story, and a video that shows the Names of martyrs.
B: Physical	B.2: Staging	7-what is the element that was responsible in narrating the story at Al-Karamah episode for you? You may choose more than one answer<br>the tanks<br>the soldiers<br>the orange tress<br>the murals<br>The explosion sound and fire<br>The songs<br>Nothing was responsible and I did not get the story	Cognitive	Based on Figure 2, most participants indicated that tanks, followed by soldiers and orange trees, were the key elements in the museum responsible for conveying the story in Al-Karamah Episode. Songs were identified as the least prominent element in the museum for narrating the story at Al- Karamah Episode.<br>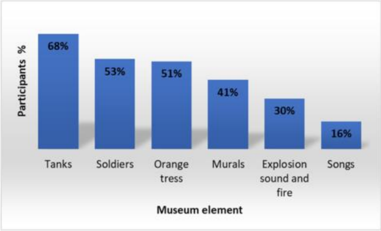
B: Physical	B.2: Staging	8-how did the orange trees affect on the scene at Al- Karamah episode?	Cognitive	When asked about the impact of orange trees on the ambiance of Al-Karamah Episode, respondents stated that their presence increased legitimacy and instilled a national spirit. Several attendees commented on how the orange trees added to the overall appeal and authenticity of the battle and events at Al-Karamah, while also conveying a profound feeling of the historical burden associated with the place. A minority of respondents, on the other hand, claimed that the presence of orange trees had little or no effect on the environment.
C: Sensorial	C1: senses	9-had you feel the heat of the battle during your visit to Al- Karamah episode?	Emotional	More than half of the participants said they felt the heat of the battle during their visit to Al- Karamah episode (43 'yes' 31 'no'),
C: Sensorial	C1: senses	10-Had you feel the heat of the Tanks?<br>○ Yes<br>○ No	Emotional	and they also felt the heat of the Tanks (47 'yes' 27 'no').
C: Sensorial	C.2: Movement	11-On the map below, kindly assign:<br>▪ if there is/are a specific position from which you observed the whole scene of the battle, kindly assign it with “X on the map. If there is no specific point, please write” No”<br>▪ kindly assign your stops at Al- karamah episode through	Cognitive	11 of 74 did not answer this question<br>7 of 63 couldn’t define a spot to see the whole story<br>8 of 63 couldn’t define a specific tank they recall

		using this symbol on the map:<br>▪ Is there a favorite tank at this section? Please encircle it, or else, write:” No”		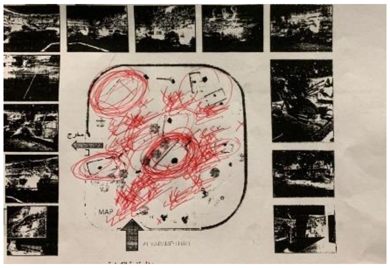<br>Most spots focused on the entrance spot, middle spot and exit spot to see the whole story. In the same distribution they also remember their stops.<br>The most tank was special is the one on the right front of the entrance<br>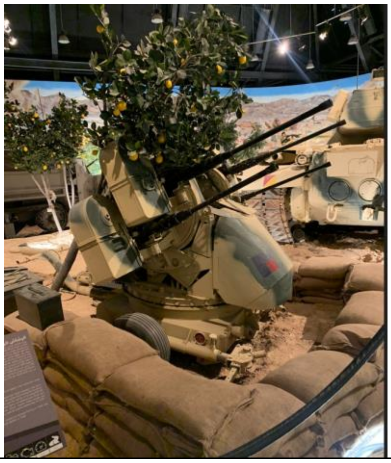
D: Emotional Experience		12-Did you relate to soldiers at Al-Karamah episode?<br>○ Yes<br>○ No	Emotional	The majority of attendees (n=50, 67.6%) said they felt connected to soldiers in Al-Karamah episode
D: Emotional Experience		13-In general, how was your feeling inside Al-Karamah episode?<br>• Glory and Pride<br>• Anger and sadness<br>• Neutral…I did not feel anything	Emotional	In terms of feelings within Al-Karamah Episode, the majority of attendees (83.8%) felt Glory and Pride, 14.9% felt nothing, and only 1.4% felt Anger and Sadness.
D: Emotional Experience		14-What did you feel while walking among the tanks in Al-Karamah Episode? You can choose more than one answer<br>• The difficulty of the war and the heat of the situation<br>• Pride and glory<br>• Neutral…I did not feel anything	Emotional	Regarding feelings while walking among the tanks in Al-Karamah Episode, more than half of the attendees felt the difficulty of the war and the heat of the situation (60.8%), 50.0% felt Glory and Pride, and only 10.8% felt nothing.
D: Emotional Experience		15- At the end of your journey in Al-	Emotional	The majority of the participants felt

		Karamah episode. You felt<br>• positive<br>• negative<br>• Neutral…I did not feel anything		positive at the end of the journey in Al-Karamah episode, 16.2% felt Neutral, and only 5.4% felt negative.
D: Emotional Experience		16- Can you please assign the above feeling if it hits your body on this graph? you may use X or circles to draw.<br>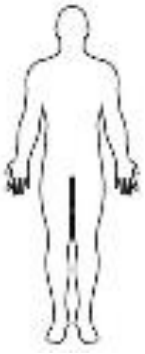	Emotional	13 out 74 did not answer the question<br>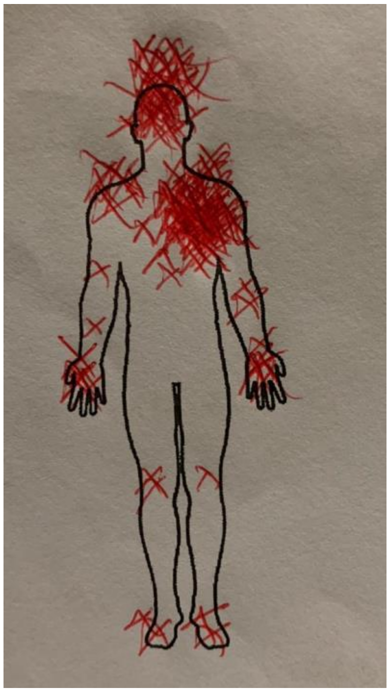<br>Most answers focused on the heart and mind<br>As if it is emotional and cognitive attached. However, it hits the heart more which gives an indication to emotional experience.
C: sensorial	C.1: Senses	17-Did the songs move you to the moment of victory?<br>Yes<br>No	Emotional	Yes 77%
C: sensorial	C.1: Senses	18-Did the presence of the speech of His Majesty King Hussein - may God have mercy on him - affect your experience at Al-Karamah Episode?<br>Yes<br>No	Emotional	77% yes
D: Emotional Experience		19-King Hussein’s speech made you feel<br>Pride and happiness<br>Anger and sadness	Emotional	The figure below shows that the majority of the participants felt with pride and happiness during listening to King Hussein’s speech in Al-Karamah Episode.

		Nothing		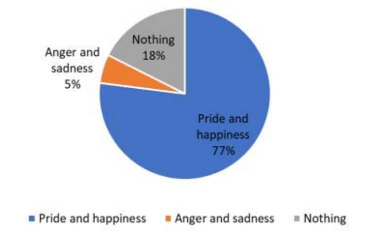
B: Physical	B.2: Staging	20-Describe one of the scenarios that you saw in Al-Karamah Episode, concerning the soldiers in the Battle of Al-Karamah.	cognitive	The answers included many scenarios which included; Prid, War, War tactics, and Soldiers' story.
B: Physical	B.2: Staging	21-Describe one of the scenarios that you saw in the Al-Karamah Episode related to the mural.	Cognitive	The answers included the following: the mural explained well what happened and very expressive
B: Physical	B.2: Staging	22-Have you realized the songs of the top Arab singers Fairooz* or Omar Al Abdallat*?<br>• Yes<br>• No<br>*Top singers whose patriotic songs are well known	Cognitive	About half of the participants realized the songs of Fairooz or Omar Al Abdallat (55.4%).
E: Immersion		25-Did you realize the existence of the other visitors in Al-Karamah episode?<br>• Yes<br>• No	Cognitive	More than half of the participants (60.8%) are aware of the presence of other visitors in Al-Karamah Episode
B: Physical	B.2 Staging	26-How would you rate the scene of Al-Karama from 5 with soldiers and tanks in terms of its reflection of the story?<br>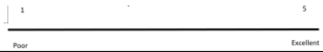	Emotional	Mean: 3.91<br>Close to excellent
E: Immersion		27-Did you feel as if you were in the battlefield or one of the soldiers while visiting Al- Karamah Episode?<br>• Yes<br>• No	Emotional	63.5 % believe they are on the battlefield or one of the soldiers while visiting Al- Karamah Episode.
Episode 6 A: Jerusalem: Bab Alwad* *Participants were shown an image of the episode				
A: narrative		28-Do you recognize the name of this section at the museum? If yes, what is it called? *	Cognitive	Out of two-thirds of the participants, 18 answered correctly, while the remaining participants did not provide an answer.
A: narrative		29-Do you know the name of the battle which happened here? *	Cognitive	Out of two-thirds of the participants (46 participants), only 20 answered correctly, while the others did not provide an answer.
A: narrative		30- How do you know the name of this area or this battle?	Cognitive	The 20 participants who answered Q29 had prior knowledge of the area and the battle. Remarkably, only

		• I studied about it<br>• I lived there<br>• I have read the sign<br>• I don’t know its name		one participant had a direct connection to the actual location in Palestine.
A: narrative		31-Can you please narrate the scene above?	Cognitive	43.2% of participants were able to accurately narrate the scene in a single sentence, identifying it as a war scene with soldiers defending their territory. However, they did not connect it to the specific battle or its actual location.
B: Physical	B.2: Staging	32-What element helped you to understand the above scene? You may choose more than one answer<br>○ The soldiers<br>○ The wall<br>○ The tank<br>○ I did not get the story	Cognitive	The figure illustrates the elements that may aid in understanding the scene. Half of the participants (50.0%) indicated that the wall helped them comprehend the scene, followed by the soldiers (43.2%), and then the tank (17.6%).<br>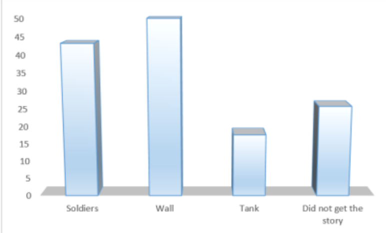
B: Physical	B.2: Staging	33- Please rate the staging of the scene above on a scale of 1 to 5, considering both design and aesthetic perspectives.<br>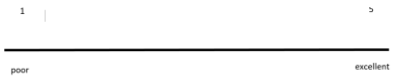 	Cognitive	From a design and aesthetic perspective, the scene received a rating of 3.95 out of 5, indicating it was considered somewhat excellent.
C: Sensorial	C.2	34-Do you want to go up the stairs to see what the soldiers are watching?<br>○ Yes<br>○ No	Cognitive	Most visitors (87.8%) expressed curiosity to climb the stairs to see what the soldiers are observing.
D: Emotional Experience		35- How did you feel in general in this section?<br>○ Victory<br>○ loss<br>○ did not feel anything	Emotional	About 64.9% felt victory, , 8% loss, and 27% felt nothing
D: Emotional Experience		36-How would you describe your interaction with this site of the museum?<br>Emotional-empathy<br>No interaction	Cognitive	68.9% had Emotional-empathy during visiting this section.
C: Sensorial	C.2	37-Kindly assign your stops position on the provided map using “X”. If you have not stopped, you may draw a	Cognitive	22 participants out of 74 did not stop.

		continuous line showing your walking pathway.		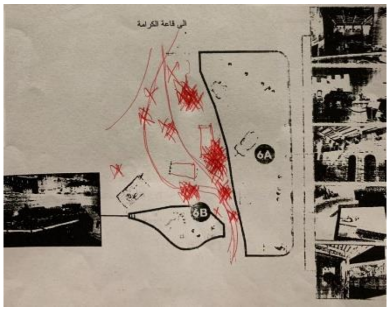<br><br>Most of the answers focused on the spot that sees the main gate of the wall as seen below<br>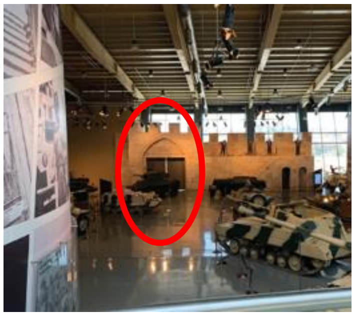<br>Another spot that reveals Al-Karamah episode (episode 8)<br>The people who passed by Episode 6A and stopped to see its wall missed the opportunity to visually see Episode 6B (Al-Latrun) because it was behind them. This resulted in a partial understanding of the entire Jerusalem area, as one of its main parts was overlooked due to the visitor's route. Consequently, the Jerusalem episode was not comprehended holistically.
E: Immersion		Did you feel that you were part of this scene?<br>• Yes<br>• No	Cognitive	80% of answers were no.
Episode 6 B: Jerusalem: Al-Latrun battle *Participants were shown an image of the episode				
A: Narrative		38-Have you spotted this area in the museum?*<br>○ Yes<br>○ No	Cognitive	More than half of the visitors reported that they spotted this area in the museum (67.6%).
A: Narrative		39-Do you know the name of this area*? Or battle? If so, Can you please narrate this scene in your own words	Cognitive	On the other hand about two-thirds of the visitors didn't know the name of this area or battle.
B: Physical	B.2: Staging	40- What element helped you to understand the story of this battle? You	Cognitive	The figure shows the elements that may help in understanding the story

		can choose more than one answer<br>○ The soldier<br>○ The Mural<br>○ The cannon<br>○ Nothing- I did not get the story		of the battle. About 36.5% of the participants reported that the soldiers helped them to understand the story, followed by the Mural (33.8%), and then the cannon (27.0%).<br>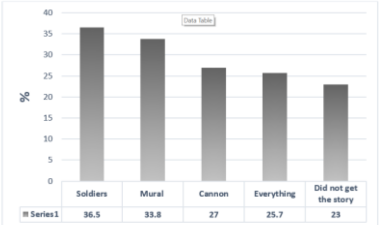
E: Immersion		41-Did you feel that you were part of this battle?<br>○ Yes<br>○ No	Emotional	About half of the visitors feel that they were part of this battle.
D: Emotional Experience		42-How did you feel in general in this section?<br>○ Anger because of loss<br>○ Pride and glory because of victory<br>○ I don't feel anything	Emotional	Only 8.1% felt Anger because of loss, and 48.6% felt nothing.
D: Emotional Experience		43-Rate your empathy with the soldier in this battle<br>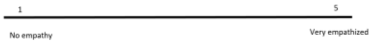 	Emotional	The mean rating of empathy with the soldier in this battle was 3.3 out of 5, indicating that visitors felt somewhat empathized.
B: Physical	B.2: Staging	44-How do you rate the mural scene with the soldier from a design and aesthetic point of view?<br>○ The scene is perfect and complete and I understood the story<br>○ The scene is perfect and complete. However, I did not get the story	Cognitive	Around two-thirds (71.6%) reported that the scene was perfect and complete. However, they did not grasp the story, as the mural relates to the castle of Al-Latrun, which was not identified by visitors.
B: Physical	B.2 Staging	45-Do you realize the relationship according the provided map , do you realize the relationship between the two sectios (6A and 6B)?<br>○ Yes<br>○ No	Cognitive	Yes: 33.8%<br>No: 66.2%<br>66% of the participants did not recognize that both sections are related to one place which is Jerusalem
A: Narrative		46-the authors told the participants the story of Al-Latrun battle. Accordingly, they were re-asked again:<br>Is this information affective to draw your attention to the wall and the battle and to get you engaged?		Yes (86.5%)<br>No: 13.5 %

	• Yes<br>• No		
